# Supplementary material for: Cross-sectional study using primary care and cancer registration data to investigate patients with cancer presenting with non-specific symptoms
Source: BMJ Open. 2020 Jan 10;10(1):e033008. doi: 10.1136/bmjopen-2019-033008 (PMC6955554; doi:10.1136/bmjopen-2019-033008)
Supplement: Supplementary data [file bmjopen-2019-033008supp001.pdf]

Cross-sectional study using primary care and cancer registration data to investigate cancer patients presenting with non-specific symptoms.

<u>Supplementary Information</u>

Table S1: Site groupings: ICD10 (International Classification of Disease version 10) codes

Site name	ICD10 codes (3 digit)
Brain & Central Nervous System (CNS)	C69-C72; C47
Breast	C50
Colorectal	C17-C21; C26
Gynaecology	C51-C58
Haematology	C81-C85; C88; C90-C93; C95-C96
Head & Neck	C00-C14; C30-C32; C73
Lung	C33-C34; C37-C38; C45
Sarcoma	C40-C41; C46; C48-C49
Skin	C43
Upper Gastrointestinal (GI)	C15-C16; C22-C25
Urology	C60-C68
Other	C74-C80

Pearson C, *et al. BMJ Open* 2020; 10:e033008. doi: 10.1136/bmjopen-2019-033008

Table S2: Regression analysis results – associations (Odds Ratios – OR) with having a long primary care interval

	All		NSCS		Non-NSCS	
	Unadjusted OR (95%CI)	Adjusted OR (95%CI)	Unadjusted OR (95%CI)	Adjusted OR (95%CI)	Unadjusted OR (95%CI)	Adjusted OR (95%CI)
**NSCS**						
NSCS	**1.59 (1.46-1.73)**	**1.24 (1.12-1.36)**				
Non-NSCS	Reference	Reference				
**Age group**						
0-24	1.34 (0.97-1.86)	0.95 (0.67-1.34)	1.91 (0.91-4.00)	1.56 (0.71-3.44)	1.23 (0.86-1.78)	0.79 (0.53-1.17)
25-44	**0.78 (0.67-0.92)**	1.08 (0.90-1.29)	1.22 (0.81-1.85)	1.37 (0.88-2.12)	**0.76 (0.64-0.91)**	1.02 (0.84-1.25)
45-59	**0.88 (0.78-0.98)**	1.08 (0.95-1.21)	1.06 (0.83-1.37)	1.15 (0.89-1.50)	**0.85 (0.75-0.97)**	1.05 (0.92-1.21)
60-69	Reference	Reference	Reference	Reference	Reference	Reference
70-79	1.04 (0.94-1.14)	0.98 (0.88-1.09)	0.96 (0.78-1.18)	0.94 (0.76-1.16)	1.05 (0.94-1.17)	0.98 (0.87-1.11)
80+	1.10 (0.99-1.21)	0.97 (0.87-1.08)	0.93 (0.76-1.15)	0.86 (0.69-1.07)	1.12 (0.99-1.26)	1.01 (0.88-1.15)
**Sex**						
Male	Reference	Reference	Reference	Reference	Reference	Reference
Female	**0.79 (0.74-0.85)**	1.09 (1.00-1.19)	1.05 (0.90-1.22)	1.12 (0.95-1.32)	**0.72 (0.67-0.78)**	1.08 (0.98-1.20)
**Deprivation**						
1 - least deprived	Reference	Reference	Reference	Reference	Reference	Reference
2	**1.12 (1.01-1.25)**	1.12 (1.00-1.25)	1.04 (0.82-1.31)	1.04 (0.82-1.32)	**1.14 (1.01-1.28)**	1.14 (1.00-1.30)
3	1.09 (0.98-1.21)	1.05 (0.94-1.17)	1.07 (0.85-1.35)	1.05 (0.82-1.32)	1.08 (0.96-1.22)	1.04 (0.92-1.18)
4	**1.18 (1.06-1.32)**	1.10 (0.98-1.23)	1.18 (0.93-1.49)	1.15 (0.90-1.46)	**1.16 (1.02-1.31)**	1.08 (0.94-1.23)
5 - most deprived	**1.19 (1.06-1.34)**	1.08 (0.96-1.23)	1.18 (0.92-1.51)	1.13 (0.88-1.45)	**1.17 (1.03-1.34)**	1.06 (0.92-1.22)
**Comorbidities**						
0	Reference	Reference	Reference	Reference	Reference	Reference
1	**1.20 (1.10-1.32)**	**1.13 (1.02-1.25)**	**1.27 (1.03-1.55)**	**1.38 (1.11-1.72)**	**1.17 (1.05-1.30)**	1.06 (0.94-1.19)
2	**1.21 (1.09-1.33)**	1.11 (0.99-1.25)	1.18 (0.95-1.46)	**1.30 (1.03-1.65)**	**1.19 (1.06-1.33)**	1.06 (0.93-1.21)
3+	**1.46 (1.31-1.61)**	**1.26 (1.11-1.42)**	**1.29 (1.04-1.61)**	**1.44 (1.12-1.84)**	**1.48 (1.32-1.66)**	**1.20 (1.04-1.38)**
**Route to diagnosis**						
Emergency presentation	**3.06 (2.78-3.37)**	**2.08 (1.87-2.32)**	**1.76 (1.46-2.13)**	**1.66 (1.36-2.03)**	**3.40 (3.03-3.82)**	**2.23 (1.95-2.54)**
GP referral	**1.78 (1.63-1.95)**	**1.44 (1.31-1.58)**	**1.32 (1.07-1.62)**	**1.28 (1.04-1.58)**	**1.84 (1.66-2.04)**	**1.48 (1.33-1.64)**

Pearson C, *et al. BMJ Open* 2020; 10:e033008. doi: 10.1136/bmjopen-2019-033008

Inpatient	**2.65 (2.03-3.45)**	**2.04 (1.55-2.67)**	1.26 (0.74-2.13)	1.18 (0.69-2.03)	**3.15 (2.31-4.28)**	**2.40 (1.75-3.30)**
Outpatient	**3.40 (2.95-3.91)**	**2.47 (2.13-2.86)**	**1.88 (1.40-2.52)**	**1.71 (1.26-2.31)**	**3.82 (3.25-4.48)**	**2.73 (2.31-3.23)**
Two Week Wait	Reference	Reference	Reference	Reference	Reference	Reference
Unknown	**2.90 (2.32-3.62)**	**2.69 (2.13-3.39)**	**1.99 (1.20-3.31)**	**1.85 (1.10-3.12)**	**3.08 (2.41-3.95)**	**2.91 (2.24-3.76)**
**Stage**						
1	Reference	Reference	Reference	Reference	Reference	Reference
2	**0.88 (0.78-0.99)**	0.93 (0.81-1.05)	0.89 (0.65-1.22)	0.94 (0.68-1.30)	**0.83 (0.73-0.95)**	0.93 (0.81-1.07)
3	**1.20 (1.07-1.36)**	0.96 (0.85-1.09)	0.97 (0.72-1.30)	1.04 (0.76-1.41)	**1.17 (1.03-1.34)**	0.94 (0.81-1.09)
4	**1.76 (1.58-1.95)**	1.06 (0.94-1.20)	1.21 (0.93-1.58)	1.14 (0.86-1.51)	**1.76 (1.56-1.98)**	1.03 (0.90-1.18)
Unknown/other	**1.81 (1.63-2.02)**	1.08 (0.95-1.23)	1.31 (1.00-1.72)	1.22 (0.90-1.64)	**1.79 (1.59-2.01)**	1.04 (0.90-1.20)
**Site**						
Brain & CNS[$]	**2.86 (2.12-3.84)**	**2.27 (1.65-3.12)**	**17.42 (2.29-132.50)**	**12.57 (1.61-97.95)**	**3.11 (2.26-4.27)**	**2.19 (1.55-3.08)**
Breast	**0.33 (0.28-0.39)**	**0.47 (0.39-0.56)**	1.66 (0.63-4.40)	1.58 (0.58-4.31)	**0.38 (0.32-0.46)**	**0.48 (0.39-0.60)**
Colorectal	Reference	Reference	Reference	Reference	Reference	Reference
Gynaecology	**0.75 (0.63-0.89)**	**0.81 (0.67-0.98)**	0.88 (0.63-1.22)	0.84 (0.59-1.20)	0.81 (0.65-1.01)	0.82 (0.65-1.04)
Haematology	**1.46 (1.25-1.70)**	**1.38 (1.17-1.62)**	**1.39 (1.08-1.79)**	1.24 (0.94-1.63)	**1.64 (1.35-2.01)**	**1.49 (1.20-1.83)**
Head & neck	0.95 (0.78-1.16)	1.10 (0.89-1.35)	1.63 (0.51-5.17)	1.38 (0.43-4.44)	1.12 (0.89-1.40)	1.15 (0.91-1.46)
Lung	**1.89 (1.65-2.16)**	**1.82 (1.58-2.11)**	**1.66 (1.31-2.11)**	**1.55 (1.21-1.99)**	**2.22 (1.86-2.65)**	**1.98 (1.64-2.38)**
Other	**1.59 (1.26-2.00)**	1.26 (0.98-1.62)	1.24 (0.87-1.77)	0.97 (0.65-1.44)	**1.99 (1.46-2.71)**	**1.57 (1.13-2.20)**
Sarcoma	**1.52 (1.08-2.12)**	**1.61 (1.13-2.28)**	1.16 (0.55-2.47)	1.08 (0.50-2.33)	**1.85 (1.26-2.71)**	**1.85 (1.24-2.76)**
Skin	**0.42 (0.35-0.52)**	**0.57 (0.46-0.71)**	1.16 (0.07-18.63)	1.33 (0.08-21.86)	**0.50 (0.40-0.63)**	**0.60 (0.47-0.75)**
Upper GI[@]	1.13 (0.98-1.31)	1.05 (0.90-1.23)	1.15 (0.92-1.43)	1.04 (0.83-1.31)	1.17 (0.96-1.43)	1.08 (0.87-1.33)
Urology	1.02 (0.90-1.16)	**1.26 (1.10-1.45)**	1.26 (0.96-1.65)	**1.39 (1.04-1.85)**	1.16 (0.98-1.37)	**1.29 (1.08-1.53)**

* Adjusted model – adjusted for NSCS (in overall cohort only), age, sex, deprivation, comorbidities, route, stage, site

[$] - Central Nervous System

[@] - Upper Gastrointestinal

Pearson C, *et al. BMJ Open* 2020; 10:e033008. doi: 10.1136/bmjopen-2019-033008
