# Supplementary material for: Cross-sectional study using primary care and cancer registration data to investigate patients with cancer presenting with non-specific symptoms
Source: BMJ Open. 2020 Jan 10;10(1):e033008. doi: 10.1136/bmjopen-2019-033008 (PMC6955554; doi:10.1136/bmjopen-2019-033008)
Supplement: Author's manuscript [file bmjopen-2019-033008.draft_revisions.pdf]

BMJ Open is committed to open peer review. As part of this commitment we make the peer review history of every article we publish publicly available.

When an article is published we post the peer reviewers' comments and the authors' responses online. We also post the versions of the paper that were used during peer review. These are the versions that the peer review comments apply to.

The versions of the paper that follow are the versions that were submitted during the peer review process. They are not the versions of record or the final published versions. They should not be cited or distributed as the published version of this manuscript.

BMJ Open is an open access journal and the full, final, typeset and author-corrected version of record of the manuscript is available on our site with no access controls, subscription charges or pay-per-view fees (http://bmjopen.bmj.com).

If you have any questions on BMJ Open's open peer review process please email info.bmjopen@bmj.com

BMJ Open

Cross-sectional study using primary care and cancer registration data to investigate cancer patients presenting with non-specific symptoms

Journal:	*BMJ Open*
Manuscript ID	bmjopen-2019-033008
Article Type:	Research
Date Submitted by the Author:	16-Jul-2019

Complete List of Authors:	Pearson, Clare; Cancer Research UK, Policy and Information; National Cancer Registration and Analysis Service,<br>Poirier, Veronique; Cancer Research UK, Policy and Information<br>Fitzgerald, Karen; Cancer Research UK, Policy & Information<br>Rubin, Greg; Royal Victoria Infirmary, Institute of Health and Society, Newcastle University<br>Hamilton, Willie; University of Exeter, Primary Care, Medical School

Keywords:	ONCOLOGY, Epidemiology < ONCOLOGY, PRIMARY CARE

SCHOLARONE™
Manuscripts

Cross-sectional study using primary care and cancer registration data to investigate cancer patients presenting with non-specific symptoms.

Clare Pearson*[a,b], Veronique Poirier[a], Karen Fitzgerald[a], Greg Rubin[c], Willie Hamilton[d]

[a] Cancer Research UK

[b] National Cancer Registration and Analysis Service, Public Health England

[c] Institute of Health and Society, University of Newcastle

[d] University of Exeter Medical School (Primary Care)

***Corresponding author:** Clare Pearson, Policy and Information Directorate, Cancer Research UK, The Angel Building, 407 St John Street, London, EC1V 4AD. clare.pearson@cancer.org.uk

Keywords: Cancer, Diagnostic intervals, Early diagnosis, Non-specific symptoms

Word count:

Abstract: 294
Article Summary: 163
Article: 2,598

Abstract

Introduction

Patients presenting to primary care with site-specific alarm symptoms can be referred onto urgent suspected cancer pathways, whereas those with non-specific symptoms currently have no dedicated referral routes leading to delays in cancer diagnosis and poorer outcomes. Pilot Multidisciplinary Diagnostic Centres (MDCs) provide a referral route for such patients in England.

Objectives

This work aimed to use linked primary care and cancer registration data to describe the problems in and after primary care facing patients similar to those being referred into MDCs.

Methods

This cross-sectional study linked primary care data from the National Cancer Diagnosis Audit (NCDA) to national cancer registration and Route to Diagnosis records. Patient symptoms recorded in the NCDA were used to allocate patients to one of two groups – those presenting with symptoms mirroring referral criteria of MDCs and those with at least one site-specific alarm symptom. Descriptive analyses compared the two groups and regression analysis by group investigated associations with long primary care intervals (PCIs).

Results

Patients with MDC referral criteria symptoms were more likely to be diagnosed at later stage (32% stage 4, compared with 21% in non-MDC group) and via an emergency presentation (24% vs 16%). There were more multiple pre-referral GP consultations (59% vs 43%) and primary care led diagnostics (blood tests: 57% vs 35%) in the MDC group. Associations between longer PCIs and late stage (stage 4 vs 1) were detected, with adjusted Odds Ratios (OR) of 1.31[95% CI: 1.17-1.46], and by different diagnostic routes (OR: 1.66[1.51-1.82] - routine vs urgent GP referral). Sensitivity analysis by MDC

For peer review only - http://bmjopen.bmj.com/site/about/guidelines.xhtml

group showed similar associations by diagnostic route, but no significant associations by stage in the MDC group.

Conclusions

Differences in the diagnostic pathway show that patients with symptoms mirroring the MDC referral criteria need a new referral pathway.

Article Summary: Strengths and limitations of this study

- The key strength of this study is the linkage of cancer registrations to primary care data which enhances our understanding of the diagnostic pathway for cancer patients.
- Comparing different groups of patients based on their presenting symptoms in primary care enables a focus on patients similar to those being referred into England's pilot Multidisciplinary Diagnostic Centres (MDCs) who do not currently have an urgent referral pathway.
- A limitation of this study is that it does not compare patients presenting with symptoms that would trigger an urgent referral under the NICE (National Institute for Health and Care Excellence) guidelines to those who experience non-urgent referral symptoms, instead it mirrors the referral criteria of the MDCs to describe diagnostic pathways for these patients.
- Symptoms recorded in our study in primary care relied on accurate reporting.
- Not all MDC referral criteria could be included in our study, notably GP intuition/patient concern which were not recordable in the data.

For peer review only

For peer review only - http://bmjopen.bmj.com/site/about/guidelines.xhtml

Introduction

Earlier detection of cancer improves clinical outcomes and quality of life for cancer patients, through improved treatment options and increased likelihood of survival. Patients in England who present to their general practitioner (GP) with site-specific 'alarm' symptoms are likely to be referred via an urgent pathway under the 'Suspected Cancer: recognition and referral' guidelines from the National Institute for Health and Care Excellence (NICE) (1) and are subsequently monitored within the Cancer Waiting Times timeframes (2). This 'Two Week Wait' (TWW) diagnostic route refers to the 14 days target between referral and seeing a specialist (3). However, previous studies have identified large proportions of cancer patients diagnosed without having alarm symptoms in general practice in Denmark (52%) (4), with similar proportions in the UK (5) and higher rates in Norway (60%) (6,7).

In England, for patients who present without such site-specific alarm symptoms warranting a TWW referral, it can be challenging for GPs to select the most appropriate referral pathway. Examples of such non-specific but concerning symptoms (NSCS) include unexplained weight loss, fatigue and some types of abdominal pain (8). Patients presenting with NSCS could therefore experience repeated referrals to different secondary care departments before a cancer diagnosis is confirmed. Overall, this could lead to unstructured and prolonged diagnostic pathways, which could negatively impact on outcomes, such as poorer survival (9), stage (9) or patient experience (10). Patients with less alarm/specific symptoms are more likely to be diagnosed via emergency presentations (11), which is itself associated with poorer outcomes (12).

A potential solution for these patients has been recently trialled and evaluated by the Accelerate, Coordinate and Evaluate (ACE) Programme, which is a joint initiative between Cancer Research UK, Macmillan Cancer Support and NHS England. The Programme aims to achieve earlier cancer diagnosis. Wave 2 of the ACE Programme has examined whether Multidisciplinary Diagnostic Centres (MDCs) can support earlier and faster diagnosis of cancers/non-cancer conditions for patients with no clear urgent diagnostic pathway. The MDCs aim to provide comprehensive diagnostics under the care of the same team to provide a more rapid diagnosis of cancer and other conditions (13). Similar programmes have been implemented in Denmark (14,15) and Sweden (16).

The National Cancer Diagnosis Audit (NCDA) (17) provides rich primary care data for cancer patients diagnosed in 2014 and can be linked to cancer registrations and other health datasets held at the National Cancer Registration and Analysis Service (NCRAS), Public Health England to build a picture of the diagnostic pathway. Symptoms at presentation to the GP are recorded in the NCDA enabling comparison of groups of patients presenting with different kinds of symptoms.

The aim of this particular study was to use linked primary care data to provide understanding of any unmet need for patients similar to those being referred into the MDCs.

Methods

Datasets

Cancer registrations from 2014 in England were sent to participating GP surgeries where primary care information was collated to create the National Cancer Diagnosis Audit (NCDA). This included dates of presentation and referral, symptoms at presentation, primary care led investigations, and many others (17). The Routes to Diagnosis (RtD) dataset (18) is generated at NCRAS, using several linked health datasets to determine the most likely diagnostic route, including emergency presentations, inpatient, TWW and routine GP referrals.

For peer review only - http://bmjopen.bmj.com/site/about/guidelines.xhtml

Data linkage

The NCDA and RtD datasets were linked with cancer registration data at tumour level using tumour ID.

Allocation to symptom groups

There are 84 distinct symptoms listed in the NCDA. To reflect patients being referred into the MDCs, the patients in the linked dataset were allocated to one of two groups depending on the symptoms at presentation to the GP within the NCDA dataset. The symptoms used to allocate patients were derived from the combined referral criteria to the MDCs and common presenting symptoms of the MDCs and are listed in table 1.

Table 1: List of Non-Specific but Concerning Symptoms (NSCS) – MDC referral criteria and common presenting symptoms

Distention	
Pallor	
Abdominal pain (upper, lower, NOS*)	* NOS (not otherwise specified)
Bowel habit change	
Constipation	
Diarrhoea	
Nausea and/or vomiting	
Fatigue	
Weight loss	
Back pain	
New onset diabetes	
Lymphadenopathy (generalised & localised)	
Deep vein thrombosis	
Loss of appetite	
Chest pain	
Chest infection	
Jaundice†	† For local reasons this specific symptom was a MDC referral symptom in one project

To be allocated to the NSCS group, patients could only have symptom(s) listed in table 1. If a patient had symptom(s) listed in table 1 but in addition had one or more other symptom, usually triggering a TWW referral (e.g. a lump, bleeding), they were allocated to the non-NSCS group. Patients with unknown symptoms in the NCDA were excluded; this could be due to screening (where GPs could not enter symptoms) or the symptoms were not known to the GP.

The primary care interval was defined as the time from first relevant presentation to the GP to when the patient is referred into secondary care (19) and is recorded for patients in the NCDA.

Exclusions

Patients with no symptom information were not allocated to one of the symptom-based groups and therefore excluded (n=3,844) (Figure 1). We also excluded cancers diagnosed via death certificate (n=13) or screening (n=14) in the RtD. Patients with primary care intervals of a negative value (n=218) or over 730 days (n=80) were excluded as in previous methodology (17,20).

For peer review only - http://bmjopen.bmj.com/site/about/guidelines.xhtml

Statistical analysis

Comorbidities were recorded in the NCDA and categorised by patient in our analysis. Frequencies and proportions of patients by different socio-demographic and disease characteristics were described in the NSCS and non-NSCS groups. Differences between proportions in the two groups by characteristic were assessed by Chi^2 tests. The primary care intervals were also described by characteristic and NSCS/non-NSCS group. Based on the distribution of the PCI and clinical advice, the interval was divided into less than and including 28 days and over 28 days for regression analysis to denote a longer PCI. We used multivariable logistic regression with longer PCI as the outcome variable with socio-demographic and disease characteristics as explanatory variables (age group, sex, comorbidities, deprivation, route to diagnosis and stage). The regression analysis was also stratified by NSCS/non-NSCS group. Analyses were conducted in Stata 15.1.

Patient and public involvement

Data for this study are based on information collected by the NHS. Patients and the public were not involved in the development of this study.

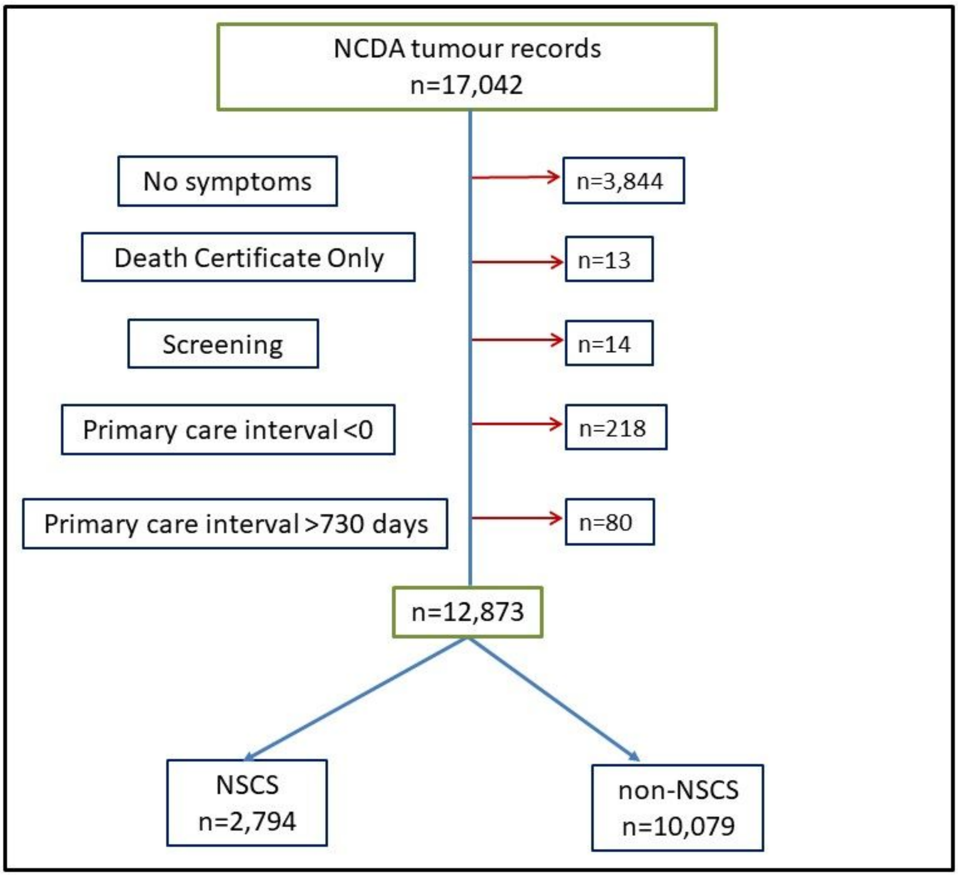

Figure 1: Data exclusions and allocation to analysis groups

Results

There were 17,042 cancers records in the NCDA linked to cancer registration data. Following exclusions, 2,794 (22% of remaining NCDA cohort) patients had only MDC symptoms recorded and were allocated to the NSCS group. 10,079 (78%) patients had at least one non-NSCS symptom and were therefore allocated to the non-NSCS group (Figure 1). Table 2 shows frequencies of patients in

For peer review only - http://bmjopen.bmj.com/site/about/guidelines.xhtml

each of the NSCS and non-NSCS groups by socio-demographic and disease characteristics and the PCI for each group and characteristic, and table 3 describes pathway characteristics and corresponding PCIs.

For peer review only

For peer review only - http://bmjopen.bmj.com/site/about/guidelines.xhtml

Table 2: Characteristics of NSCS and non-NSCS patients & PCIs

				Primary care interval (days)	
	NSCS	Non-NSCS	P value+	NSCS	Non-NSCS
	n (%)	n (%)		Median (IQR)	Median (IQR)
Total	2,794 (21.7)	10,079 (78.3)		12 (1-39)	3 (0-23)
**Age group**			<0.001		
0-24	33 (1.18)	123 (1.22)		0 (0-47.5)	5 (0-29)
25-44	105 (3.76)	712 (7.06)		13 (0-45)	0 (0-15)
45-59	387 (13.85)	1,865 (18.50)		13 (0-42)	1 (0-19)
60-69	645 (23.09)	2,439 (24.20)		14 (1-41)	5 (0-28)
70-79	827 (29.60)	2,759 (27.37)		13 (1-42)	4 (0-24)
80+	797 (28.53)	2,181 (21.64)		9 (1-31)	3 (0-22)
**Sex**			0.331		
Male	1,410 (50.47)	5,191 (51.50)		11 (0-36)	7 (0-29)
Female	1,384 (49.53)	4,888 (48.50)		13 (1-43)	0 (0-15)
**Deprivation quintile**			0.050		
1 – least deprived	557 (19.94)	2,227 (22.10)		11 (1-36)	3 (0-23)
2	599 (21.44)	2,182 (21.65)		13 (1-41)	3 (0-26)
3	595 (21.30)	2,182 (21.65)		11.5 (1-35)	2 (0-23)
4	567 (20.29)	1,900 (18.85)		11 (0-42.5)	2 (0-21)
5 - most deprived	476 (17.04)	1,588 (15.76)		13 (0-41.5)	3 (0-24)
**Comorbidities**			<0.001		
0	661 (23.66)	2,787 (27.65)		9 (0-37)	1 (0-21)
1	853 (30.53)	2,993 (29.70)		13 (1-43)	3 (0-25)
2	678 (24.27)	2,289 (22.71)		13 (1-39)	3 (0-23)
3+	602 (21.55)	2,010 (19.94)		13 (1-39)	4 (0-28)
**Diagnostic route**			<0.001		
Emergency presentation	949 (33.97)	1,623 (16.10)		5 (0-27)	5 (0-26)
GP referral	681 (24.37)	2,509 (24.89)		14 (1-44)	7 (0-31)
TWW	802 (28.70)	4,782 (47.45)		14 (3-42)	1 (0-17)
Inpatient elective	60 (2.15)	171 (1.70)		9.5 (0-45)	5 (0-48)
Outpatient	235 (8.41)	724 (7.18)		12 (0-45)	5 (0-29)
Unknown	67 (2.40)	270 (2.68)		10.5 (0-27)	8 (0-45)
**Stage**			<0.001		
1	290 (10.38)	2,573 (25.53)		13 (0-42)	0 (0-15)
2	357 (12.78)	1,828 (18.14)		10 (0-39)	0 (0-14)
3	444 (15.89)	1,516 (15.04)		12 (1-38)	5 (0-28)
4	897 (32.10)	2,094 (20.78)		14 (1-42)	8 (0-35)
Unknown	806 (28.85)	2,068 (20.52)		10 (0.5-37)	5 (0-28)

+ - significance test of proportions in NSCS/non-NSCS by characteristic

The NSCS group were older with the median (Inter-Quartile Range – IQR) age in the NSCS group of 72 years (63-81) and 69 (58-78) in non-NSCS. There were higher proportions of the NSCS group in the two oldest age groups. All factors in table 2 (apart from sex) show that there were significantly different proportions in NSCS compared with non-NSCS. Higher proportions of the NSCS group resided in more deprived areas and had more comorbidities. There were higher proportions of the NSCS group diagnosed via emergency presentation and lower proportions in the TWW diagnostic route. Excluding unknown stage, 33% of NSCS group were diagnosed at stages 1 or 2, compared with 55% in the non-

For peer review only - http://bmjopen.bmj.com/site/about/guidelines.xhtml

For peer review only

NSCS group, with correspondingly higher proportions of NSCS patients diagnosed at late stage (excluding unknown: stages 3 and 4: NSCS 67%, non-NSCS 45%).

PCIs were consistently longer in the NSCS group in all categories of characteristics and at all levels with a wider IQR, with only a couple of exceptions. Patients diagnosed via the emergency presentation route had the same PCI for both groups. Patients under 25 years in non-NSCS had longer PCIs than the NSCS group. Primary care intervals were longer for every stage of disease at diagnosis in the NSCS group.

Table 3: Pathway characteristics of NSCS and non-NSCS patients & primary care intervals

				Primary care interval	
	NSCS	Non-NSCS	P value+	NSCS	Non-NSCS
	n (%)	n (%)		Median (IQR)	Median (IQR)
**Number of consultations before referral***			<0.001		
1	792 (28.35)	4,362 (43.28)		0 (0-4)	0 (0-1)
2	742 (26.35)	2,197 (21.80)		12 (5-25)	14 (6-30)
3	356 (12.74)	882 (8.75)		28 (11-59)	30 (14-58.5)
4	196 (7.02)	460 (4.56)		40 (21-73)	42 (20-88)
5+	353 (12.63)	761 (7.55)		58 (33-134)	76 (33-148)
**Primary care led investigations**					
Blood tests	1,589 (56.87)	3,510 (34.82)		15 (4-45)	14 (3-41)
Urinary	21 (0.75)	175 (1.74)		35 (8-69)	14 (5-38)
Imaging	930 (33.29)	2,176 (21.59)		23 (7-55)	21 (6-50.5)
Imaging - X-Ray	393 (14.07)	1,330 (13.20)		21 (6-55)	18 (5-50)
Imaging - CT	123 (4.40)	226 (2.24)		27 (7-53.5)	35 (16-76.5)
Imaging - Ultrasound	513 (18.36)	818 (8.12)		28 (10-58)	24 (8-51)
Imaging - MRI	32 (1.15)	61 (0.61)		35 (9-60)	33.5 (11-98)
Endoscopy	95 (3.40)	152 (1.51)		38 (6-94)	11.5 (0-50)
Endoscopy - Upper GI	52 (1.86)	102 (1.01)		41 (7-95)	14 (0-50)
Endoscopy - Colon	45 (1.61)	44 (0.44)		42 (5-108)	7 (0-73)
Other	314 (11.24)	904 (8.97)		20 (6-50)	17 (4-49)
None	712 (25.48)	4,544 (45.08)		0 (0-12)	0 (0-2)
**Avoidable delays?**			<0.001		
Yes	738 (26.41)	2,215 (21.98)		30 (6-85)	21 (0-70)
No	1,825 (65.32)	7,256 (71.99)		7 (0-25)	1 (0-14)
Unknown	231 (8.27)	608 (6.03)		15 (0-42)	6 (0-36)
* excluding 0 consultations					

+ - significance test of proportions in NSCS/non-NSCS by characteristic

There were higher proportions of patients with multiple consultations and more primary care led investigations (apart from urinary investigations) in the NSCS group, there was also a higher proportion where the GP felt that there was an avoidable delay to their diagnosis in this group (table 3). Patients could have more than one investigation, so significance testing was not undertaken on this element in table 3, however, the other characteristics in table 3 had statistically significantly different proportions in NSCS/non-NSCS with all p values <0.001.

The PCI was longer in the non-NSCS group for patients presenting more than twice before referral. There was variation in PCI by group for investigations, with longer intervals in the NSCS group for most

For peer review only - http://bmjopen.bmj.com/site/about/guidelines.xhtml

tests, the exception being CT. PCIs were longer in those with an avoidable delay in the NSCS group and longer in both groups where there was an avoidable delay to diagnosis.

Table 4 shows unadjusted and adjusted odds ratios (ORs) of having a long primary care interval for the entire NCDA cohort and stratified by NSCS/non-NSCS group. In the entire NCDA cohort, after adjustment for age, sex, deprivation, comorbidities, route and stage, being in the NSCS group was associated with having a longer primary care interval. Females had reduced odds of having a long primary care interval when compared with males. Compared with the least deprived, patients in the two most deprived quintiles had higher odds of having a longer interval. Compared with TWW, all other routes had higher odds of having a longer interval. Compared with stage 1, patients diagnosed at stage 4 had higher odds of having a longer interval. Higher comorbidity score was also associated with a longer primary care interval. There were no significant associations between age group and primary care intervals.

When stratified by NSCS/non-NSCS, the decreased odds for females of having longer primary care interval was only evident in the non-NSCS group. The significant associations by deprivation in the entire cohort were not evident in either group. Comorbidity remained significantly associated with longer primary care intervals only in the highest category in the non-NSCS group. The ORs by diagnosis route remained significantly associated with the primary care interval and demonstrated a similar pattern for both groups. Stage was not significantly associated with longer primary care intervals in the NSCS group but remained significant in the non-NSCS group.

For peer review only

For peer review only - http://bmjopen.bmj.com/site/about/guidelines.xhtml

Table 4: Regression analysis results – associations (Odds Ratios – OR) with having a long primary care interval

	Logistic regression		NSCS		Non-NSCS	
	Unadjusted OR (95%CI)	Adjusted OR (95%CI)	Unadjusted OR (95%CI)	Adjusted OR (95%CI)	Unadjusted OR (95%CI)	Adjusted OR (95%CI)
**NSCS**						
NSCS	**1.59 (1.46-1.73)**	**1.28 (1.17-1.40)**				
Non-NSCS	Reference	Reference				
**Age group**						
0-24	1.34 (0.97-1.86)	1.11 (0.79-1.56)	1.91 (0.91-4.00)	1.88 (0.88-4.03)	1.23 (0.86-1.78)	0.96 (0.65-1.42)
25-44	0.78 (0.67-0.92)	0.93 (0.78-1.11)	1.22 (0.81-1.86)	1.33 (0.87-2.05)	0.76 (0.64-0.91)	0.89 (0.73-1.08)
45-59	0.88 (0.78-0.98)	0.97 (0.87-1.10)	1.06 (0.83-1.37)	1.12 (0.86-1.45)	0.85 (0.75-0.97)	0.95 (0.83-1.09)
60-69						
70-79	1.04 (0.94-1.14)	0.98 (0.88-1.08)	0.96 (0.78-1.18)	0.92 (0.75-1.14)	1.05 (0.94-1.17)	0.99 (0.88-1.11)
80+	1.10 (0.99-1.21)	0.94 (0.84-1.05)	0.93 (0.76-1.15)	0.84 (0.67-1.04)	1.12 (1.00-1.27)	0.97 (0.85-1.10)
**Sex**						
Male	Reference	Reference	Reference	Reference	Reference	Reference
Female	**0.79 (0.74-0.85)**	**0.86 (0.80-0.92)**	1.05 (0.90-1.22)	1.05 (0.90-1.22)	**0.72 (0.67-0.78)**	**0.82 (0.75-0.89)**
**Deprivation**						
1 - least deprived	Reference	Reference	Reference	Reference	Reference	Reference
2	**1.12 (1.01-1.25)**	**1.13 (1.01-1.26)**	1.04 (0.82-1.31)	1.05 (0.83-1.33)	**1.14 (1.01-1.28)**	**1.14 (1.01-1.30)**
3	1.09 (0.98-1.21)	1.07 (0.96-1.20)	1.07 (0.85-1.35)	1.08 (0.85-1.36)	1.08 (0.96-1.22)	1.07 (0.94-1.21)
4	**1.18 (1.06-1.32)**	**1.14 (1.01-1.27)**	1.18 (0.93-1.49)	1.16 (0.91-1.47)	**1.16 (1.02-1.31)**	1.13 (0.99-1.28)
5 - most deprived	**1.19 (1.06-1.33)**	**1.15 (1.02-1.29)**	1.18 (0.92-1.51)	1.14 (0.89-1.46)	**1.17 (1.03-1.34)**	1.14 (0.99-1.31)
**Comorbidities**						
0	Reference	Reference	Reference	Reference	Reference	Reference
1	**1.20 (1.10-1.32)**	**1.15 (1.04-1.28)**	**1.27 (1.03-1.55)**	**1.39 (1.12-1.73)**	**1.17 (1.05-1.30)**	1.09 (0.97-1.23)
2	**1.21 (1.09-1.33)**	**1.14 (1.02-1.28)**	1.18 (0.95-1.46)	**1.33 (1.05-1.68)**	**1.19 (1.06-1.33)**	1.10 (0.96-1.25)
>=3	**1.46 (1.31-1.61)**	**1.30 (1.15-1.47)**	**1.29 (1.04-1.61)**	**1.46 (1.14-1.88)**	**1.48 (1.32-1.66)**	**1.25 (1.09-1.44)**
**Route to diagnosis**						
Emergency presentation	**3.06 (2.78-3.37)**	**2.50 (2.25-2.77)**	**1.76 (1.46-2.13)**	**1.65 (1.35-2.01)**	**3.40 (3.03-3.82)**	**2.83 (2.50-3.21)**

For peer review only - http://bmjopen.bmj.com/site/about/guidelines.xhtml

GP referral	**1.78 (1.63-1.95)**	**1.66 (1.51-1.82)**	**1.32 (1.07-1.62)**	**1.28 (1.04-1.58)**	**1.84 (1.66-2.04)**	**1.72 (1.55-1.91)**
Inpatient	**2.65 (2.04-3.46)**	**2.36 (1.81-3.08)**	1.26 (0.74-2.13)	1.22 (0.72-2.07)	**3.15 (2.31-4.28)**	**2.83 (2.08-3.87)**
Other outpatient	**3.40 (2.95-3.91)**	**3.07 (2.66-3.55)**	**1.88 (1.40-2.53)**	**1.77 (1.31-2.39)**	**3.82 (3.25-4.48)**	**3.48 (2.96-4.10)**
TWW	Reference	Reference	Reference	Reference	Reference	Reference
Unknown	**2.90 (2.32-3.62)**	**2.77 (2.21-3.47)**	**1.99 (1.20-3.31)**	**1.88 (1.12-3.16)**	**3.08 (2.41-3.95)**	**2.98 (2.32-3.84)**
**Stage**						
1	Reference	Reference	Reference	Reference	Reference	Reference
2	0.88 (0.78-0.99)	0.89 (0.78-1.00)	0.89 (0.65-1.22)	0.90 (0.66-1.24)	0.83 (0.73-0.95)	0.88 (0.77-1.00)
3	**1.20 (1.07-1.36)**	1.12 (0.99-1.27)	0.97 (0.72-1.30)	0.99 (0.73-1.34)	**1.17 (1.03-1.34)**	1.12 (0.98-1.29)
4	**1.76 (1.58-1.95)**	**1.34 (1.20-1.50)**	1.21 (0.93-1.58)	1.16 (0.89-1.53)	**1.76 (1.56-1.98)**	**1.36 (1.20-1.54)**
Unknown/other	**1.81 (1.63-2.02)**	**1.31 (1.17-1.46)**	**1.31 (1.00-1.72)**	1.19 (0.90-1.57)	**1.79 (1.59-2.01)**	**1.30 (1.15-1.48)**

* Adjusted model – adjusted for NSCS (in overall cohort only), age, sex, deprivation, comorbidities, route, stage

For peer review only

For peer review only - http://bmjopen.bmj.com/site/about/guidelines.xhtml

Discussion

Summary of main findings

This large study used existing data to examine patients with cancer who could have been eligible for referral to a MDC. It showed clear differences in such patients and those eligible for urgent suspected cancer referral, whereby the former experienced longer primary care intervals, had more primary care interactions, were more likely to be diagnosed at later stage and via emergency presentation.

Strengths and limitations

The use of primary care data linked with cancer registrations enables a detailed picture of the diagnostic pathway for cancer patients. Symptoms recorded in the NCDA provide a basis for examining different groups of patients.

The allocation into the NSCS and non-NSCS groups by symptom is a proxy for distinguishing between alarm and non-alarm symptoms in the NICE referral guidelines. True separation between alarm symptoms warranting a referral onto an urgent referral pathway and vague symptoms which do not is difficult, especially for symptoms where there are more than one recommendation depending on other symptoms and patient characteristics (such as appetite loss, with five different recommendations and weight loss with 13 recommendations). This required additional information about patient characteristics is not all available in the NCDA. This study, however, instead reflected the MDC referral criteria and common presenting symptoms recorded at MDCs. Indeed, the aim of this work was to provide evidence of the possible problems facing patients similar to those potentially eligible to go through the MDCs.

Not all MDC referral criteria were recorded in the NCDA, including GP intuition or patient/family concern, though it is unlikely that the inclusion of this would have changed the allocation to symptom groups. Additionally, not all of the non-specific symptoms in our allocation list are truly low risk, with jaundice being the most debatable, instead being an alarm symptom for particular cancers, and it is genuinely high risk for pancreatic cancer, though much lower for other cancers (21,22). Symptoms recorded in the NCDA were not necessarily complete and relied on accurate recording in primary care systems for those completing the NCDA to extract (17).

Comparisons with the literature

Previous work using the NCDA has shown significant variation in the patient interval length by different abdominal symptoms (23), and cohort studies have shown similar variation by symptoms for colorectal (24), lung (25) and pancreatic cancer patients (26). Other studies using linked primary care data have found longer diagnostic intervals (from symptom presentation to diagnosis) for those presenting with non-alarm symptoms (5,27,28) when compared with alarm symptoms patients. A previous study examining lung cancer patients found longer primary care intervals for patients presenting with vague symptoms (29).

Previous work on primary care intervals have shown variation in primary care intervals by cancer site (17,30,31), yet only one has focused on different symptom profiles of lung cancer patients (29).

Our study adds to this body of literature by examining the diagnostic pathways of patients diagnosed with a wide range of cancers presenting with non-specific but concerning symptoms.

Interpretation and implications

For peer review only - http://bmjopen.bmj.com/site/about/guidelines.xhtml

The lack of specific referral pathways for patients who present with non-specific symptoms is well described (32,33). Our work explains the problems facing patients who presented with non-specific symptoms, similar to those to be referred into the MDCs, with longer primary care intervals and more primary care interactions. The higher proportion of late stage disease in those presenting with NSCS may relate to the passing of time until the symptoms became more pronounced, leading to a cancer diagnosis at a later stage of disease, possibly via an emergency – which we show that NSCS patients are also more likely to experience. These patients have longer time intervals before referral to secondary care indicating the lack of clear referral route onto a specific urgent cancer referral pathway.

The overall sex difference in the regression results, where females are less likely to have longer intervals, is only evident in the non-NSCS group. This could be driven by breast cancer, predominantly diagnosed after a woman presents to their GP with a lump and are referred under the TWW pathway, though adjustment was made by route to diagnosis in the analysis.

The association between late stage disease and longer primary care intervals was not evident in the NSCS group, probably due, in part, to the significantly higher proportions of emergency presentations in this group, who have shorter intervals and tend to be at a later stage of disease (Table 2).

We have demonstrated patients presenting with NSCS who would fulfil the criteria for MDC referral take longer to reach a diagnosis than those likely to be referred on an urgent suspected cancer pathway. They also have higher proportions of late stage/emergency presentations. This study does not show that MDCs can expedite diagnosis, but that there is a problem, for which MDCs may be the answer.

Conclusion

Using national linked data, we have demonstrated that patients presenting with NSCS experienced longer time intervals before diagnosis, were more likely to be diagnosed via an emergency and at a later stage of disease, all of which are associated with poorer outcomes. An alternative diagnostic referral pathway for these patients should therefore be considered.

For peer review only

For peer review only - http://bmjopen.bmj.com/site/about/guidelines.xhtml

References

1. National Institute for Health and Care Excellence. Suspected cancer: recognition and referral. NICE guideline (NG12) [Accessed: 5 Nov 2018]. 2015. Available from: https://www.nice.org.uk/guidance/ng12

2. NHS Digital. Cancer Waiting Times Monitoring Dataset [Accessed 15 Jun 2018]. Available from: https://digital.nhs.uk/services/systems-and-service-delivery/screening-services/cancer-waiting-times

3. Cancer Research UK. Cancer waiting times definitions by country. 2015 [Accessed 5 Nov 2018]. Available from: http://www.cancerresearchuk.org/sites/default/files/cs_report_cwt.pdf

4. Ingeman ML, Christensen MB, Bro F, Knudsen ST, Vedsted P. The Danish cancer pathway for patients with serious non-specific symptoms and signs of cancer–a cross-sectional study of patient characteristics and cancer probability. BMC Cancer. 2015 May 20;15(1):421.

5. Neal R, Din N, Hamilton W, Ukoumunne O, Carter B, Stapley S, et al. Comparison of cancer diagnostic intervals before and after implementation of NICE guidelines: analysis of data from the UK General Practice Research Database. Br J Cancer. 2014;110(3):584.

6. Scheel BI, Ingebrigtsen SG, Thorsen T, Holtedahl K. Cancer suspicion in general practice: the role of symptoms and patient characteristics, and their association with subsequent cancer. Br J Gen Pract J R Coll Gen Pract. 2013 Sep;63(614):e627–35.

7. Scheel BI, Hart B, Ingebrigtsen SG, Thorsen T, Holtedahl K. Frequency of 'warning signs of cancer' in Norwegian general practice, with prospective recording of subsequent cancer. Fam Pract. 2012 Oct 24;30(2):153–60.

8. Koo MM, Hamilton W, Walter FM, Rubin GP, Lyratzopoulos G. Symptom Signatures and Diagnostic Timeliness in Cancer Patients: A Review of Current Evidence. Neoplasia. 2018 Feb;20(2):165–74.

9. Neal RD, Tharmanathan P, France B, Din NU, Cotton S, Fallon-Ferguson J, et al. Is increased time to diagnosis and treatment in symptomatic cancer associated with poorer outcomes? Systematic review. Br J Cancer. 2015 Mar 31;112(s1):S92–107.

10. Mendonca SC, Abel GA, Saunders CL, Wardle J, Lyratzopoulos G. Pre-referral general practitioner consultations and subsequent experience of cancer care: evidence from the English Cancer Patient Experience Survey. Eur J Cancer Care (Engl). 2016;25(3):478–90.

11. Renzi C, Lyratzopoulos G, Card T, Chu TPC, Macleod U, Rachet B. Do colorectal cancer patients diagnosed as an emergency differ from non-emergency patients in their consultation patterns and symptoms? A longitudinal data-linkage study in England. Br J Cancer. 2016 Aug 18;115:866.

12. McPhail S, Elliss-Brookes L, Shelton J, Ives A, Greenslade M, Vernon S, et al. Emergency presentation of cancer and short-term mortality. Br J Cancer. 2013;109(8):2027.

13. Nicholson BD, Oke J, Friedemann Smith C, Phillips J-A, Lee J, Abel L, et al. The Suspected CANcer (SCAN) pathway: protocol for evaluating a new standard of care for patients with non-specific symptoms of cancer. BMJ Open. 2018 Jan 1;8(1):e018168.

For peer review only - http://bmjopen.bmj.com/site/about/guidelines.xhtml

14. Ingeman ML, Christensen MB, Bro F, Knudsen ST, Vedsted P. The Danish cancer pathway for patients with serious non-specific symptoms and signs of cancer-a cross-sectional study of patient characteristics and cancer probability. BMC Cancer. 2015 May 20;15:421–421.

15. Moseholm E, Lindhardt BØ. Patient characteristics and cancer prevalence in the Danish cancer patient pathway for patients with serious non-specific symptoms and signs of cancer—A nationwide, population-based cohort study. Cancer Epidemiol. 2017 Oct 1;50:166–72.

16. Stenman E, Palmér K, Rydén S, Sävblom C, Svensson I, Rose C, et al. Diagnostic spectrum and time intervals in Sweden's first diagnostic center for patients with nonspecific symptoms of cancer. Acta Oncol. 2019 Jan 11;1–10.

17. Swann R, McPhail S, Witt J, Shand B, Abel GA, Hiom S, et al. Diagnosing cancer in primary care: results from the National Cancer Diagnosis Audit. Br J Gen Pract 2017 68(666) e63-e72

18. Elliss-Brookes L, McPhail S, Ives A, Greenslade M, Shelton J, Hiom S, et al. Routes to diagnosis for cancer - determining the patient journey using multiple routine data sets. Br J Cancer. 2012;107. Available from: http://dx.doi.org/10.1038/bjc.2012.408

19. Weller D, Vedsted P, Rubin G, Walter FM, Emery J, Scott S, et al. The Aarhus statement: improving design and reporting of studies on early cancer diagnosis. Br J Cancer. 2012;106. Available from: http://dx.doi.org/10.1038/bjc.2012.68

20. Lyratzopoulos G, Abel G, McPhail S, Neal R, Rubin G. Measures of promptness of cancer diagnosis in primary care: secondary analysis of national audit data on patients with 18 common and rarer cancers. Br J Cancer. 2013;108(3):686.

21. Stapley S, Peters T, Neal R, Rose P, Walter F, Hamilton W. The risk of pancreatic cancer in symptomatic patients in primary care: a large case–control study using electronic records. Br J Cancer. 2012;106(12):1940.

22. Taylor A, Stapley S, Hamilton W. Jaundice in primary care: a cohort study of adults aged> 45 years using electronic medical records. Fam Pract. 2012;29(4):416–20.

23. Koo MM, von Wagner C, Abel GA, McPhail S, Hamilton W, Rubin GP, et al. The nature and frequency of abdominal symptoms in cancer patients and their associations with time to help-seeking: evidence from a national audit of cancer diagnosis. J Public Health. 2018 Jan 27; Available from: http://dx.doi.org/10.1093/pubmed/fdx188

24. Walter FM, Emery JD, Mendonca S, Hall N, Morris HC, Mills K, et al. Symptoms and patient factors associated with longer time to diagnosis for colorectal cancer: results from a prospective cohort study. Br J Cancer. 2016 Aug 4;115:533.

25. Walter FM, Rubin G, Bankhead C, Morris HC, Hall N, Mills K, et al. Symptoms and other factors associated with time to diagnosis and stage of lung cancer: a prospective cohort study. Br J Cancer. 2015 Mar 31;112(Suppl 1):S6–13.

26. Walter FM, Mills K, Mendonça SC, Abel GA, Basu B, Carroll N, et al. Symptoms and patient factors associated with diagnostic intervals for pancreatic cancer (SYMPTOM pancreatic study): a prospective cohort study. Lancet Gastroenterol Hepatol. 2016 Dec 1;1(4):298–306.

For peer review only

For peer review only - http://bmjopen.bmj.com/site/about/guidelines.xhtml

27. Din NU, Ukoumunne OC, Rubin G, Hamilton W, Carter B, Stapley S, et al. Age and gender variations in cancer diagnostic intervals in 15 cancers: analysis of data from the UK Clinical Practice Research Datalink. PLoS One. 2015;10(5):e0127717.

28. Redaniel MT, Martin RM, Ridd MJ, Wade J, Jeffreys M. Diagnostic intervals and its association with breast, prostate, lung and colorectal cancer survival in England: historical cohort study using the clinical practice research datalink. PloS One. 2015;10(5):e0126608.

29. Guldbrandt LM, Fenger-Grøn M, Rasmussen TR, Jensen H, Vedsted P. The role of general practice in routes to diagnosis of lung cancer in Denmark: a population-based study of general practice involvement, diagnostic activity and diagnostic intervals. BMC Health Serv Res. 2015;15(1):21.

30. Lyratzopoulos G, Saunders CL, Abel GA, McPhail S, Neal RD, Wardle J, et al. The relative length of the patient and the primary care interval in patients with 28 common and rarer cancers. Br J Cancer. 2015 Mar 3;112:S35.

31. Baughan P, O'Neill B, Fletcher E. Auditing the diagnosis of cancer in primary care: the experience in Scotland. Br J Cancer. 2009 Dec 3;101:S87.

32. Rubin G, Berendsen A, Crawford SM, Dommett R, Earle C, Emery J, et al. The expanding role of primary care in cancer control. Lancet Oncol. 2015;16(12):1231–72.

33. Green T, Atkin K, Macleod U. Cancer detection in primary care: insights from general practitioners. Br J Cancer. 2015;112(s1):S41.

Acknowledgements

The work was undertaken as part of the CRUK-PHE partnership and the ACE Programme, which is CRUK, Macmillan cancer support and NHS England supported. The authors would also like to thank Ruth Swann & Jess Fraser (CRUK-PHE partnership), Dave Chapman (ACE Programme, CRUK) and the National Cancer Diagnosis Audit.

Data for this study is based on patient-level information collected by the NHS, as part of the care and support of cancer patients. The data are collated, maintained and quality assured by the National Cancer Registration and Analysis Service, which is part of Public Health England

Funding statement

WH is co-PI and GR steering group chair of CanTest, a Cancer Research UK Catalyst Award [C8640/A23385].

Patient consent

Not required

Ethics

This study was exempt from gaining individual consent having obtained Section 251 approval from the UK Patient Advisory Group (PIAG) (now the Confidentiality Advisory Group, CAG), under Section 251 of the NHS Act 2006 (PIAG 03(a)/2001).

Conflicts of interests

The authors declare no conflicts of interests

For peer review only - http://bmjopen.bmj.com/site/about/guidelines.xhtml

Author statement

Conception and design of the work: CP, VP and KF.

Analysis of the data: CP with input from VP

All authors made substantial contributions to the interpretation of the findings.

All authors contributed to drafting the manuscript or revising it critically for important intellectual content and approved the final version submitted.

All authors have agreed to be accountable for all aspects of the work.

For peer review only

For peer review only - http://bmjopen.bmj.com/site/about/guidelines.xhtml

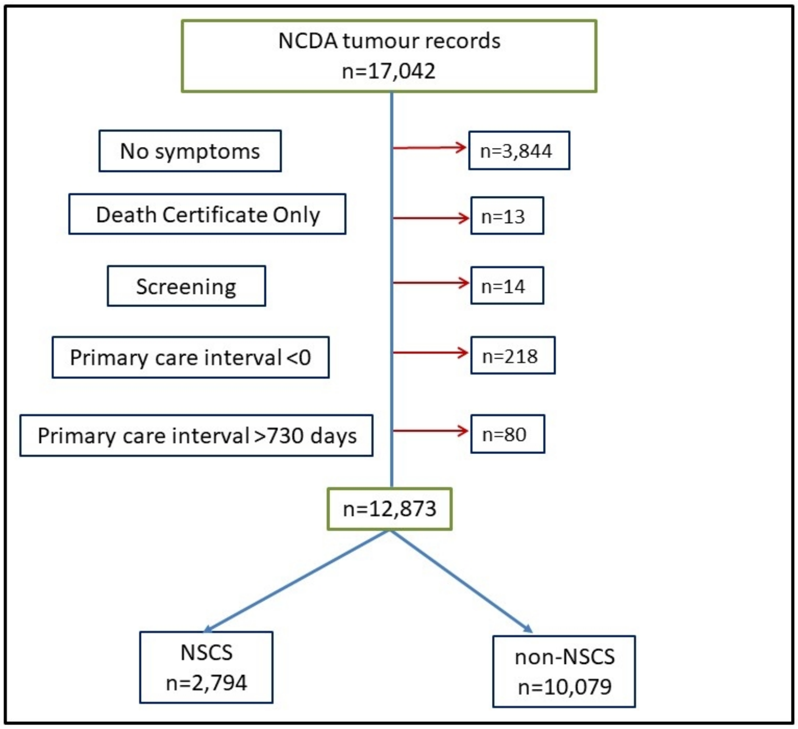

Figure 1: Data exclusions and allocation to analysis groups

81x60mm (300 x 300 DPI)

STROBE 2007 (v4) Statement—Checklist of items that should be included in reports of *cross-sectional studies*

Section/Topic	Item #	Recommendation	Reported on page #
**Title and abstract**	1	(*a*) Indicate the study's design with a commonly used term in the title or the abstract	1
		(*b*) Provide in the abstract an informative and balanced summary of what was done and what was found	1
**Introduction**			
Background/rationale	2	Explain the scientific background and rationale for the investigation being reported	3
Objectives	3	State specific objectives, including any prespecified hypotheses	3
**Methods**			
Study design	4	Present key elements of study design early in the paper	3
Setting	5	Describe the setting, locations, and relevant dates, including periods of recruitment, exposure, follow-up, and data collection	N/A
Participants	6	(*a*) Give the eligibility criteria, and the sources and methods of selection of participants	N/A
Variables	7	Clearly define all outcomes, exposures, predictors, potential confounders, and effect modifiers. Give diagnostic criteria, if applicable	4&5
Data sources/ measurement	8*	For each variable of interest, give sources of data and details of methods of assessment (measurement). Describe comparability of assessment methods if there is more than one group	3
Bias	9	Describe any efforts to address potential sources of bias	N/A in methods
Study size	10	Explain how the study size was arrived at	4
Quantitative variables	11	Explain how quantitative variables were handled in the analyses. If applicable, describe which groupings were chosen and why	5
Statistical methods	12	(*a*) Describe all statistical methods, including those used to control for confounding	5
		(*b*) Describe any methods used to examine subgroups and interactions	5
		(*c*) Explain how missing data were addressed	4
		(*d*) If applicable, describe analytical methods taking account of sampling strategy	N/A
		(*e*) Describe any sensitivity analyses	5
**Results**			

For peer review only

Participants	13*	(a) Report numbers of individuals at each stage of study—eg numbers potentially eligible, examined for eligibility, confirmed eligible, included in the study, completing follow-up, and analysed	5
		(b) Give reasons for non-participation at each stage	5
		(c) Consider use of a flow diagram	5
Descriptive data	14*	(a) Give characteristics of study participants (eg demographic, clinical, social) and information on exposures and potential confounders	7
		(b) Indicate number of participants with missing data for each variable of interest	
Outcome data	15*	Report numbers of outcome events or summary measures	7&8
Main results	16	(*a*) Give unadjusted estimates and, if applicable, confounder-adjusted estimates and their precision (eg, 95% confidence interval). Make clear which confounders were adjusted for and why they were included	10&11
		(*b*) Report category boundaries when continuous variables were categorized	7&8, 10&11
		(*c*) If relevant, consider translating estimates of relative risk into absolute risk for a meaningful time period	N/A
Other analyses	17	Report other analyses done—eg analyses of subgroups and interactions, and sensitivity analyses	9
**Discussion**			
Key results	18	Summarise key results with reference to study objectives	12
Limitations	19	Discuss limitations of the study, taking into account sources of potential bias or imprecision. Discuss both direction and magnitude of any potential bias	12
Interpretation	20	Give a cautious overall interpretation of results considering objectives, limitations, multiplicity of analyses, results from similar studies, and other relevant evidence	12&13
Generalisability	21	Discuss the generalisability (external validity) of the study results	13
**Other information**			
Funding	22	Give the source of funding and the role of the funders for the present study and, if applicable, for the original study on which the present article is based	16

*Give information separately for cases and controls in case-control studies and, if applicable, for exposed and unexposed groups in cohort and cross-sectional studies.

Note: An Explanation and Elaboration article discusses each checklist item and gives methodological background and published examples of transparent reporting. The STROBE checklist is best used in conjunction with this article (freely available on the Web sites of PLoS Medicine at http://www.plosmedicine.org/, Annals of Internal Medicine at http://www.annals.org/, and Epidemiology at http://www.epidem.com/). Information on the STROBE Initiative is available at www.strobe-statement.org.

For peer review only

BMJ Open

Cross-sectional study using primary care and cancer registration data to investigate cancer patients presenting with non-specific symptoms

Journal:	*BMJ Open*
Manuscript ID	bmjopen-2019-033008.R1
Article Type:	Original research
Date Submitted by the Author:	08-Oct-2019
Complete List of Authors:	Pearson, Clare; Cancer Research UK, Policy and Information; National Cancer Registration and Analysis Service,<br>Poirier, Veronique; Cancer Research UK, Policy and Information<br>Fitzgerald, Karen; Cancer Research UK, Policy & Information<br>Rubin, Greg; Royal Victoria Infirmary, Institute of Health and Society, Newcastle University<br>Hamilton, Willie; University of Exeter, Primary Care, Medical School
**Primary Subject Heading**:	Epidemiology
Secondary Subject Heading:	General practice / Family practice
Keywords:	ONCOLOGY, Epidemiology < ONCOLOGY, PRIMARY CARE

SCHOLARONE™
Manuscripts

Cross-sectional study using primary care and cancer registration data to investigate cancer patients presenting with non-specific symptoms.

Clare Pearson*[a,b], Veronique Poirier[a], Karen Fitzgerald[a], Greg Rubin[c], Willie Hamilton[d]

[a] Cancer Research UK

[b] National Cancer Registration and Analysis Service, Public Health England

[c] Institute of Health and Society, University of Newcastle

[d] University of Exeter Medical School (Primary Care)

***Corresponding author:** Clare Pearson, Policy and Information Directorate, Cancer Research UK, The Angel Building, 407 St John Street, London, EC1V 4AD. clare.pearson@cancer.org.uk

Keywords: Cancer, Diagnostic intervals, Early diagnosis, Non-specific symptoms

Word counts:

Abstract: 275
Article Summary: 156
Article: 2,692

Abstract

Introduction

Patients presenting to primary care with site-specific alarm symptoms can be referred onto urgent suspected cancer pathways, whereas those with non-specific symptoms currently have no dedicated referral routes leading to delays in cancer diagnosis and poorer outcomes. Pilot Multidisciplinary Diagnostic Centres (MDCs) provide a referral route for such patients in England.

Objectives

This work aimed to use linked primary care and cancer registration data to describe diagnostic pathways for patients similar to those being referred into MDCs and compare them to patients presenting with more specific symptoms.

Methods

This cross-sectional study linked primary care data from the National Cancer Diagnosis Audit (NCDA) to national cancer registration and Route to Diagnosis records. Patient symptoms recorded in the NCDA were used to allocate patients to one of two groups – those presenting with symptoms mirroring referral criteria of MDCs (Non-Specific but Concerning Symptoms (NSCS)) and those with at least one site-specific alarm symptom (non-NSCS). Descriptive analyses compared the two groups and regression analysis by group investigated associations with long primary care intervals (PCIs).

Results

Patients with NSCS were more likely to be diagnosed at later stage (32% stage 4, compared with 21% in non-NSCS) and via an emergency presentation (34% vs 16%). These patients also had more multiple pre-referral GP consultations (59% vs 43%) and primary care led diagnostics (blood tests: 57% vs 35%). Patients with NSCS had higher odds of having longer PCIs (adjusted OR: 1.24[1.11-1.36]. Lung and urological cancer patients also had higher odds of longer PCIs overall and in both groups.

For peer review only

Conclusions

Differences in the diagnostic pathway show that patients with symptoms mirroring the MDC referral criteria could benefit from a new referral pathway.

Article Summary: Strengths and limitations of this study

- The key strength of this study is the linkage of cancer registrations to primary care data enhancing our understanding of the diagnostic pathway for cancer patients.
- Comparing different groups of patients based on their presenting symptoms in primary care enables a focus on patients with symptoms similar to those being referred into England's pilot Multidisciplinary Diagnostic Centres (MDCs) who do not currently have an urgent referral pathway.
- A limitation of this study is that it does not compare patients presenting with symptoms that would trigger an urgent referral under the NICE (National Institute for Health and Care Excellence) guidelines to those who experience non-urgent referral symptoms, instead it mirrors the referral criteria of the MDCs to describe diagnostic pathways for these patients.
- Symptoms recorded in our study in primary care relied on accurate reporting.
- Not all MDC referral criteria could be included in our study, notably GP intuition/patient concern which were not recordable in the data.

For peer review only

Introduction

Earlier detection of cancer improves clinical outcomes and quality of life for cancer patients, through improved treatment options and increased likelihood of survival. Patients in England who present to their general practitioner (GP) with site-specific 'alarm' symptoms are likely to be referred via an urgent pathway under the 'Suspected Cancer: recognition and referral' guidelines from the National Institute for Health and Care Excellence (NICE) (1) and are subsequently monitored within the Cancer Waiting Times timeframes (2). This 'Two Week Wait' (TWW) diagnostic route refers to the 14 days target between referral and seeing a specialist (3). However, previous studies have identified large proportions of cancer patients diagnosed without having alarm symptoms in general practice in Denmark (52%) (4), with similar proportions in the UK (5) and higher rates in Norway (60%) (6,7).

In England, for patients who present without such site-specific alarm symptoms warranting a TWW referral, it can be challenging for GPs to select the most appropriate referral pathway. Examples of such non-specific but concerning symptoms (NSCS) include unexplained weight loss, fatigue and some types of abdominal pain (8). Patients presenting with NSCS could therefore experience repeated referrals to different secondary care departments before a cancer diagnosis is confirmed. Overall, this could lead to unstructured and prolonged diagnostic pathways, which could negatively impact on outcomes, such as poorer survival (9), later stage (9) or worse patient experience (10). Patients with less alarm/specific symptoms are more likely to be diagnosed via emergency presentations (11), which is itself associated with poorer outcomes (12). If diagnostic pathways for such patients could be improved, there is an opportunity to improve outcomes.

A potential solution for these patients has been recently trialled and evaluated by the Accelerate, Coordinate and Evaluate (ACE) Programme, which is a joint initiative between Cancer Research UK, Macmillan Cancer Support and NHS England. The Programme aims to achieve earlier cancer diagnosis. Wave 2 of the ACE Programme is evaluating whether Multidisciplinary Diagnostic Centres (MDCs) can support earlier and faster diagnosis of cancers/non-cancer conditions for patients with no clear urgent diagnostic pathway using a symptom-based approach to streamline diagnostic pathways for such patients. The MDCs aim to provide comprehensive diagnostics under the care of the same team to provide a more rapid diagnosis of cancer and other conditions (13). Similar programmes have been implemented in Denmark (4,14) and Sweden (15). There is currently little evidence of whether unmet need exists for patients in England likely to be referred into the MDCs and how their diagnostic pathways compare with patients presenting with more specific symptoms.

The National Cancer Diagnosis Audit (NCDA) (16) provides an opportunity to explore rich primary care data for cancer patients diagnosed in 2014 and when linked to cancer registrations and other health datasets held at the National Cancer Registration and Analysis Service (NCRAS), Public Health England it can build a more complete picture of diagnostic pathways. Symptoms at presentation to the GP are recorded in the NCDA enabling comparison of groups of patients presenting with different kinds of symptoms.

The aim of this particular study was to use linked primary care data to provide understanding of any unmet need for patients similar to those being referred into the MDCs and to compare them with patients presenting with more specific symptoms, specifically focusing on factors which could lead to poorer outcomes such as stage and route to diagnosis. In addition, primary care intervals (PCIs) for both groups of patients were compared.

Methods

Datasets

The National Cancer Diagnosis Audit was conducted using primary care data submitted from participating GP surgeries on a voluntary basis. Cancer registrations from 2014 in England were sent to these surgeries where primary care information was collated to create the NCDA. This included dates of presentation and referral, symptoms at presentation, primary care led investigations, and many others (16). 83% of participating surgeries completed over 95% of patient NCDA data with over 17,000 cancers submitted in total. The Routes to Diagnosis (RtD) dataset is generated at NCRAS, using several linked health datasets to determine the most likely diagnostic route (17), including emergency presentations, inpatient, TWW and routine GP referrals.

Data linkage

The NCDA and RtD datasets were linked with cancer registration data at tumour level using tumour ID. Where a patient had multiple cancers (n=385), GPs were instructed to enter the same demographic and patient details, whilst submitting symptoms, investigations and interval data for each tumour separately.

Allocation to symptom groups

There were 84 distinct symptoms listed in the NCDA. To reflect patients being referred into the MDCs, the patients in the linked dataset were allocated to one of two groups depending on the symptoms at presentation to the GP within the NCDA dataset. The symptoms used to allocate patients were derived from the combined referral criteria to the MDCs (18) and an additional common presenting symptom (Bowel habit change) of the MDCs. These symptoms are listed in table 1.

Table 1: List of Non-Specific but Concerning Symptoms (NSCS) – MDC referral criteria and common presenting symptoms

Symptom	Notes
Distention	
Pallor	
Abdominal pain (upper, lower, NOS*)	* NOS (not otherwise specified)
Bowel habit change	
Constipation	
Diarrhoea	
Nausea and/or vomiting	
Fatigue	
Weight loss	
Back pain	
New onset diabetes	
Lymphadenopathy (generalised & localised)	
Deep vein thrombosis	
Loss of appetite	
Chest pain	
Chest infection	
Jaundice†	† For local reasons this specific symptom was a referral symptom in one MDC project

To be allocated to the NSCS group, patients could only have symptom(s) listed in table 1. If a patient had symptom(s) listed in table 1 but in addition had one or more other symptom, usually triggering a TWW referral (e.g. a lump, bleeding), they were allocated to the non-NSCS group. Patients with

unknown symptoms in the NCDA were excluded; reasons for this could be due to screening (where GPs could not enter symptoms) or that the symptoms were not known to the GP.

The primary care interval (PCI) was defined as the time from first relevant presentation to the GP to when the patient is referred into secondary care (19). The first presentation date in the NCDA was completed as the date when the patient first presented with symptoms ultimately attributed by the GP to the diagnosis of cancer (16).

Cancer sites were categorised into the Cancer Waiting Times site specific grouping (20) depending on their ICD10 (International Classification of Disease v10) code. They are listed in table S1 in the Supplementary Information.

Exclusions

Patients with no symptom information could not be allocated to one of the symptom-based groups and therefore excluded (n=3,844) (Figure 1). We also excluded cancers diagnosed via death certificate (n=13) or screening (n=14) in the RtD. Patients with PCIs of a negative value (n=218) or over 730 days (n=80) were excluded as in previous methodology (16,21).

Statistical analysis

Comorbidities were recorded in the NCDA and categorised by patient in our analysis. Frequencies and proportions of patients by different socio-demographic and disease characteristics were described in the NSCS and non-NSCS groups. Differences between proportions in the two groups by characteristic were assessed by Chi^2 tests. The PCIs were also described by characteristic and NSCS/non-NSCS group. Based on the distribution of the PCI and clinical advice, the interval was divided into less than and including 28 days and over 28 days for regression analysis to denote a longer PCI. We used multivariable logistic regression with longer PCI as the outcome variable with socio-demographic and disease characteristics as explanatory variables (age group, sex, comorbidities, deprivation, route to diagnosis, cancer site and stage). The regression analysis was also stratified by NSCS/non-NSCS group. Analyses were conducted in Stata 15.1.

Patient and public involvement

Data for this study are based on information collected by the NHS. Patients and the public were not involved in the development of this study.

Figure 1: Data exclusions and allocation to symptom-based analysis groups

Results

There were 17,042 cancers records in the NCDA linked to cancer registration data. Following exclusions, 2,794 (22% of remaining NCDA cohort) patients had only MDC symptoms recorded and were allocated to the NSCS group. 10,079 (78%) patients had at least one non-NSCS symptom and were therefore allocated to the non-NSCS group (Figure 1). Table 2 shows frequencies of patients in each of the NSCS and non-NSCS groups by socio-demographic and disease characteristics along with the PCI for each group and characteristic. Table 3 describes pathway and disease characteristics and corresponding PCIs.

The NSCS group were older with the median (Inter-Quartile Range – IQR) age in the NSCS group of 72 years (63-81) and 69 (58-78) in non-NSCS. There were higher proportions of the NSCS group in the two oldest age groups. All factors in table 2 (apart from sex) show that there were significantly different

For peer review only

proportions in NSCS compared with non-NSCS. Higher proportions of the NSCS group resided in more deprived areas and had more comorbidities. There were higher proportions of the NSCS group diagnosed via emergency presentation and lower proportions in the TWW diagnostic route. Excluding unknown stage, 33% of NSCS group were diagnosed at stages 1 or 2, compared with 55% in the non-NSCS group, with correspondingly higher proportions of NSCS patients diagnosed at late stage (excluding unknown: stages 3 and 4: NSCS 67%, non-NSCS 45%).

PCIs were consistently longer in the NSCS group in all categories of characteristics and at all levels with a wider IQR, with only a couple of exceptions. Patients diagnosed via the emergency presentation route had the same PCI for both groups. Patients under 25 years in non-NSCS had longer PCIs than the NSCS group. Primary care intervals were longer for every stage of disease at diagnosis in the NSCS group.

Table 2: Characteristics of non-specific but concerning symptoms (NSCS) and non-NSCS patients & primary care intervals (median & inter-quartile range(IQR))

	Frequencies			Primary care interval (days)	
	NSCS	Non-NSCS	P value+	NSCS	Non-NSCS
	n (%)	n (%)		Median (IQR)	Median (IQR)
Total	2,794 (21.7)	10,079 (78.3)		12 (1-39)	3 (0-23)
**Age group**			<0.001		
0-24	33 (1.18)	123 (1.22)		0 (0-47.5)	5 (0-29)
25-44	105 (3.76)	712 (7.06)		13 (0-45)	0 (0-15)
45-59	387 (13.85)	1,865 (18.50)		13 (0-42)	1 (0-19)
60-69	645 (23.09)	2,439 (24.20)		14 (1-41)	5 (0-28)
70-79	827 (29.60)	2,759 (27.37)		13 (1-42)	4 (0-24)
80+	797 (28.53)	2,181 (21.64)		9 (1-31)	3 (0-22)
**Sex**			0.331		
Male	1,410 (50.47)	5,191 (51.50)		11 (0-36)	7 (0-29)
Female	1,384 (49.53)	4,888 (48.50)		13 (1-43)	0 (0-15)
**Deprivation quintile**			0.050		
1 – least deprived	557 (19.94)	2,227 (22.10)		11 (1-36)	3 (0-23)
2	599 (21.44)	2,182 (21.65)		13 (1-41)	3 (0-26)
3	595 (21.30)	2,182 (21.65)		11.5 (1-35)	2 (0-23)
4	567 (20.29)	1,900 (18.85)		11 (0-42.5)	2 (0-21)
5 - most deprived	476 (17.04)	1,588 (15.76)		13 (0-41.5)	3 (0-24)
**Comorbidities**			<0.001		
0	661 (23.66)	2,787 (27.65)		9 (0-37)	1 (0-21)
1	853 (30.53)	2,993 (29.70)		13 (1-43)	3 (0-25)
2	678 (24.27)	2,289 (22.71)		13 (1-39)	3 (0-23)
3+	602 (21.55)	2,010 (19.94)		13 (1-39)	4 (0-28)
**Diagnostic route**			<0.001		
Emergency presentation	949 (33.97)	1,623 (16.10)		5 (0-27)	5 (0-26)
GP referral	681 (24.37)	2,509 (24.89)		14 (1-44)	7 (0-31)
Two Week Wait	802 (28.70)	4,782 (47.45)		14 (3-42)	1 (0-17)
Inpatient elective	60 (2.15)	171 (1.70)		9.5 (0-45)	5 (0-48)
Outpatient	235 (8.41)	724 (7.18)		12 (0-45)	5 (0-29)
Unknown	67 (2.40)	270 (2.68)		10.5 (0-27)	8 (0-45)
**Stage**			<0.001		
1	290 (10.38)	2,573 (25.53)		13 (0-42)	0 (0-15)

2	357 (12.78)	1,828 (18.14)		10 (0-39)	0 (0-14)
3	444 (15.89)	1,516 (15.04)		12 (1-38)	5 (0-28)
4	897 (32.10)	2,094 (20.78)		14 (1-42)	8 (0-35)
Unknown/other	806 (28.85)	2,068 (20.52)		10 (0.5-37)	5 (0-28)

+ - significance test of proportions in NSCS/non-NSCS by characteristic

For peer review only

Table 3: Pathway characteristics of non-specific but concerning symptoms (NSCS) and non-NSCS patients & primary care intervals (median & inter-quartile range(IQR))

				Primary care interval (days)	
	NSCS	Non-NSCS	P value+	NSCS	Non-NSCS
	n (%)	n (%)		Median (IQR)	Median (IQR)
**Number of consultations before referral***			<0.001		
1	792 (28.35)	4,362 (43.28)		0 (0-4)	0 (0-1)
2	742 (26.35)	2,197 (21.80)		12 (5-25)	14 (6-30)
3	356 (12.74)	882 (8.75)		28 (11-59)	30 (14-58.5)
4	196 (7.02)	460 (4.56)		40 (21-73)	42 (20-88)
5+	353 (12.63)	761 (7.55)		58 (33-134)	76 (33-148)
**Primary care led investigations**					
Blood tests	1,589 (56.87)	3,510 (34.82)		15 (4-45)	14 (3-41)
Urinary	21 (0.75)	175 (1.74)		35 (8-69)	14 (5-38)
Imaging	930 (33.29)	2,176 (21.59)		23 (7-55)	21 (6-50.5)
Imaging - X-Ray	393 (14.07)	1,330 (13.20)		21 (6-55)	18 (5-50)
Imaging - CT	123 (4.40)	226 (2.24)		27 (7-53.5)	35 (16-76.5)
Imaging - Ultrasound	513 (18.36)	818 (8.12)		28 (10-58)	24 (8-51)
Imaging - MRI	32 (1.15)	61 (0.61)		35 (9-60)	33.5 (11-98)
Endoscopy	95 (3.40)	152 (1.51)		38 (6-94)	11.5 (0-50)
Endoscopy - Upper GI@	52 (1.86)	102 (1.01)		41 (7-95)	14 (0-50)
Endoscopy - Colon	45 (1.61)	44 (0.44)		42 (5-108)	7 (0-73)
Other	314 (11.24)	904 (8.97)		20 (6-50)	17 (4-49)
None	712 (25.48)	4,544 (45.08)		0 (0-12)	0 (0-2)
**Avoidable delays?**			<0.001		
Yes	738 (26.41)	2,215 (21.98)		30 (6-85)	21 (0-70)
No	1,825 (65.32)	7,256 (71.99)		7 (0-25)	1 (0-14)
Unknown	231 (8.27)	608 (6.03)		15 (0-42)	6 (0-36)
**Cancer site**			<0.001		
Brain & CNS$	16 (0.57)	208 (2.06)		64 (35-99)	3 (0-16)
Breast	17 (0.61)	1,614 (16.01)		15 (3-33)	0 (0-0)
Colorectal	804 (28.78)	817 (8.11)		8 (0-36)	3 (0-25)
Gynaecology	179 (6.41)	634 (6.29)		14 (3-35)	1 (0-19)
Haematology	338 (12.10)	782 (7.76)		12 (1-38)	10 (1-35)
Head & Neck	12 (0.43)	527 (5.23)		18 (0-45)	3 (0-28)
Lung	423 (15.14)	1,427 (14.16)		14 (1-45)	14 (2-45)
Sarcoma	28 (1.00)	121 (1.20)		12 (1-43)	12.5 (0-46.5)
Skin	<5 (<1%)	742 (7.36)		N/A	0 (0-2)
Upper GI@	551 (19.72)	750 (7.44)		10 (1-36)	5 (0-33)
Urology	279 (9.99)	2,256 (22.38)		15 (3-43)	10 (1-31)
Other	145 (5.19)	201 (1.99)		9 (0-35)	6 (0-32)

+ - significance test of proportions in NSCS/non-NSCS by characteristic

* excluding 0 consultations

$ Central Nervous System

@ Upper Gastrointestinal

There were higher proportions of patients with multiple consultations and more primary care led investigations (apart from urinary investigations) in the NSCS group, there was also a higher proportion where the GP felt that there was an avoidable delay to their diagnosis in this group (table

For peer review only

3). Patients could have more than one investigation, so significance testing was not undertaken on this element in table 3, however, the other characteristics in table 3 had statistically significantly different proportions in NSCS/non-NSCS with all p values <0.001. There were higher proportions of breast, head & neck, brain & CNS and urological cancers in the non-NSCS group. Median PCIs were the same in both groups for lung cancer, similar for sarcoma, but longer in the NSCS group for all other groupings.

The PCI was longer in the non-NSCS group for patients presenting more than twice before referral. There was variation in PCI by group for investigations, with longer intervals in the NSCS group for most tests, the exception being CT. PCIs were longer in those with an avoidable delay in the NSCS group and longer in both groups where there was an avoidable delay to diagnosis.

Table S2 (Supplementary Information) shows unadjusted and adjusted odds ratios (ORs) of having a long PCI for the entire NCDA cohort (n=12,873) and stratified by NSCS (n=2,974) /non-NSCS (n=10,079) group. In the entire NCDA cohort, after adjustment for age, sex, deprivation, comorbidities, route, stage and site, being in the NSCS group was associated with having a longer PCI (adjusted OR[95% Confidence Intervals: 1.24[1.12-1.36]. Compared with TWW, all other routes had higher odds of longer intervals. Higher comorbidity scores were also associated with longer PCIs. Compared with colorectal cancers, patients with haematological, lung, sarcoma, brain & CNS and urological cancers were more likely to have longer PCIs. When stratified by NSCS/non-NSCS, comorbidity remained significantly associated with longer PCIs for all scores in NSCS and only in the highest category in the non-NSCS group. Associations by diagnostic route remained significant with a similar pattern for both groups, with lower odds in the NSCS group. Breast cancers had lower odds of having longer PCI in only in the non-NSCS group and only lung, brain & CNS and urological cancers had higher odds in both groups of having longer PCIs compared with colorectal cancer.

Discussion

Summary of main findings

This large study used existing data to examine patients with cancer who could have been eligible for referral to a MDC. It showed clear differences in such patients and those eligible for urgent suspected cancer referral, whereby the former experienced longer PCIs, had more primary care interactions, were more likely to be diagnosed at later stage and via emergency presentation.

Strengths and limitations

The use of primary care data linked with cancer registrations enables a detailed picture of the diagnostic pathway for cancer patients. Symptoms recorded in the NCDA provide a basis for examining different groups of patients.

The allocation into the NSCS and non-NSCS groups by symptom is a proxy for distinguishing between alarm and non-alarm symptoms in the NICE referral guidelines. An analysis of symptom groups with a true separation between alarm symptoms warranting a referral onto an urgent referral pathway and vague symptoms which do not, would be very difficult. This is especially the case for symptoms where there are more than one recommendation depending on other symptoms and patient characteristics (such as appetite loss, with five different recommendations and weight loss with 13 recommendations). Such analysis would also require patient characteristic information which is not all available in the NCDA linked data. This study, however, focused on the MDC referral criteria and common presenting symptoms recorded at MDCs. Indeed, the aim of this work was to provide

For peer review only

evidence of the possible diagnostic problems facing patients similar to those potentially eligible to go through the MDCs.

Not all MDC referral criteria were recorded in the NCDA, including GP intuition or patient/family concern, though it is unlikely that the inclusion of this would have changed the allocation to symptom groups. Additionally, not all of the non-specific symptoms in our allocation list are truly low risk, with jaundice being the most debatable, instead being an alarm symptom for particular cancers, and it is genuinely high risk for pancreatic cancer, though much lower for other cancers (22,23). Symptoms recorded in the NCDA were not necessarily complete and relied on accurate recording in primary care systems for those completing the NCDA to extract (16).

Comparisons with the literature

Previous work using the NCDA has shown significant variation in the patient interval (symptom onset to presentation) by different abdominal symptoms (24), and cohort studies have shown similar variation of different diagnostic intervals by symptoms for colorectal (25), lung (26) and pancreatic cancer patients (27). Other studies using linked primary care data have found longer diagnostic intervals (from symptom presentation to diagnosis) for those presenting with non-alarm symptoms (5,28,29) when compared with alarm symptoms patients. A previous study examining lung cancer patients found longer PCIs for patients presenting with vague symptoms (30).

Previous work on PCIs have shown variation in these intervals by cancer site (16,31,32), yet only one has focused on different symptom profiles of lung cancer patients (30).

Our study adds to this body of literature by examining the diagnostic pathways of patients diagnosed in England with a wide range of cancers presenting with non-specific but concerning symptoms.

Interpretation and implications

The lack of specific referral pathways for patients who present with non-specific symptoms is well described (33,34). Our work explains the problems facing patients who presented with non-specific symptoms, similar to those to be referred into the MDCs, with longer PCIs more primary care interactions. The higher proportion of late stage disease in those presenting with NSCS may relate to the passing of time until the symptoms became more pronounced, leading to a cancer diagnosis at a later stage of disease, possibly via an emergency – which we show that NSCS patients are also more likely to experience. These patients have longer time intervals before referral to secondary care indicating the lack of clear referral route onto a specific urgent cancer referral pathway.

The association between certain sites (lung, urology) and longer PCIs was evident in both groups and overall, probably due, in part, to presenting symptoms.

We have demonstrated patients presenting with NSCS who would fulfil the criteria for MDC referral take longer to reach a diagnosis than those likely to be referred on an urgent suspected cancer pathway. They also have higher proportions of late stage/emergency presentations. This study does not show that MDCs can expedite diagnosis, but indicates the problems facing patients diagnosed with cancer who present with non-specific symptoms, for which MDCs may be the answer. The results of the MDC evaluations will be published separately.

Conclusion

Using national linked data, we have demonstrated that patients presenting with NSCS experienced longer time intervals before diagnosis, were more likely to be diagnosed via an emergency and at a

For peer review only

later stage of disease, all of which are associated with poorer outcomes. An alternative diagnostic referral pathway for these patients should therefore be considered.

For peer review only

References

1. National Institute for Health and Care Excellence. Suspected cancer: recognition and referral. NICE guideline (NG12) [Accessed: 5 Nov 2018]. 2015. Available from: https://www.nice.org.uk/guidance/ng12

2. NHS Digital. Cancer Waiting Times Monitoring Dataset [Accessed 15 Jun 2018]. Available from: https://digital.nhs.uk/services/systems-and-service-delivery/screening-services/cancer-waiting-times

3. Cancer Research UK. Cancer waiting times definitions by country. 2015 [Accessed 5 Nov 2018]. Available from: http://www.cancerresearchuk.org/sites/default/files/cs_report_cwt.pdf

4. Ingeman ML, Christensen MB, Bro F, Knudsen ST, Vedsted P. The Danish cancer pathway for patients with serious non-specific symptoms and signs of cancer–a cross-sectional study of patient characteristics and cancer probability. BMC Cancer. 2015 May 20;15(1):421.

5. Neal R, Din N, Hamilton W, Ukoumunne O, Carter B, Stapley S, et al. Comparison of cancer diagnostic intervals before and after implementation of NICE guidelines: analysis of data from the UK General Practice Research Database. Br J Cancer. 2014;110(3):584.

6. Scheel BI, Ingebrigtsen SG, Thorsen T, Holtedahl K. Cancer suspicion in general practice: the role of symptoms and patient characteristics, and their association with subsequent cancer. Br J Gen Pract J R Coll Gen Pract. 2013 Sep;63(614):e627–35.

7. Scheel BI, Hart B, Ingebrigtsen SG, Thorsen T, Holtedahl K. Frequency of 'warning signs of cancer' in Norwegian general practice, with prospective recording of subsequent cancer. Fam Pract. 2012 Oct 24;30(2):153–60.

8. Koo MM, Hamilton W, Walter FM, Rubin GP, Lyratzopoulos G. Symptom Signatures and Diagnostic Timeliness in Cancer Patients: A Review of Current Evidence. Neoplasia. 2018 Feb;20(2):165–74.

9. Neal RD, Tharmanathan P, France B, Din NU, Cotton S, Fallon-Ferguson J, et al. Is increased time to diagnosis and treatment in symptomatic cancer associated with poorer outcomes? Systematic review. Br J Cancer. 2015 Mar 31;112(s1):S92–107.

10. Mendonca SC, Abel GA, Saunders CL, Wardle J, Lyratzopoulos G. Pre-referral general practitioner consultations and subsequent experience of cancer care: evidence from the English Cancer Patient Experience Survey. Eur J Cancer Care (Engl). 2016;25(3):478–90.

11. Renzi C, Lyratzopoulos G, Card T, Chu TPC, Macleod U, Rachet B. Do colorectal cancer patients diagnosed as an emergency differ from non-emergency patients in their consultation patterns and symptoms? A longitudinal data-linkage study in England. Br J Cancer. 2016 Aug 18;115:866.

12. McPhail S, Elliss-Brookes L, Shelton J, Ives A, Greenslade M, Vernon S, et al. Emergency presentation of cancer and short-term mortality. Br J Cancer. 2013;109(8):2027.

13. Nicholson BD, Oke J, Friedemann Smith C, Phillips J-A, Lee J, Abel L, et al. The Suspected CANcer (SCAN) pathway: protocol for evaluating a new standard of care for patients with non-specific symptoms of cancer. BMJ Open. 2018 Jan 1;8(1):e018168.

For peer review only

14. Moseholm E, Lindhardt BØ. Patient characteristics and cancer prevalence in the Danish cancer patient pathway for patients with serious non-specific symptoms and signs of cancer—A nationwide, population-based cohort study. Cancer Epidemiol. 2017 Oct 1;50:166–72.

15. Stenman E, Palmér K, Rydén S, Sävblom C, Svensson I, Rose C, et al. Diagnostic spectrum and time intervals in Sweden's first diagnostic center for patients with nonspecific symptoms of cancer. Acta Oncol. 2019 Jan 11;1–10.

16. Swann R, McPhail S, Witt J, Shand B, Abel GA, Hiom S, et al. Diagnosing cancer in primary care: results from the National Cancer Diagnosis Audit. Br J Gen Pract 2017 68(666) e63-e72

17. Elliss-Brookes L, McPhail S, Ives A, Greenslade M, Shelton J, Hiom S, et al. Routes to diagnosis for cancer - determining the patient journey using multiple routine data sets. Br J Cancer. 2012;107. Available from: http://dx.doi.org/10.1038/bjc.2012.408

18. ACE Programme. Key messages from the evaluation of the Multidisciplinary Diagnostic Centres (MDC): a new approach to the diagnosis of cancer. 2019 [Accessed 16 Sept 2019]. Available from: https://www.cancerresearchuk.org/sites/default/files/ace_mdc_report_may_2019_1.1.pdf

19. Weller D, Vedsted P, Rubin G, Walter FM, Emery J, Scott S, et al. The Aarhus statement: improving design and reporting of studies on early cancer diagnosis. Br J Cancer. 2012;106. Available from: http://dx.doi.org/10.1038/bjc.2012.68

20. Public Health England. Cancer Outcomes and Services Data set. Pathology user guide, v. 4.0. 2019 [Accessed 16 Sept 2019] p 71-92. Available from: http://www.ncin.org.uk/collecting_and_using_data/data_collection/cosd_downloads_v9

21. Lyratzopoulos G, Abel G, McPhail S, Neal R, Rubin G. Measures of promptness of cancer diagnosis in primary care: secondary analysis of national audit data on patients with 18 common and rarer cancers. Br J Cancer. 2013;108(3):686.

22. Stapley S, Peters T, Neal R, Rose P, Walter F, Hamilton W. The risk of pancreatic cancer in symptomatic patients in primary care: a large case–control study using electronic records. Br J Cancer. 2012;106(12):1940.

23. Taylor A, Stapley S, Hamilton W. Jaundice in primary care: a cohort study of adults aged> 45 years using electronic medical records. Fam Pract. 2012;29(4):416–20.

24. Koo MM, von Wagner C, Abel GA, McPhail S, Hamilton W, Rubin GP, et al. The nature and frequency of abdominal symptoms in cancer patients and their associations with time to help-seeking: evidence from a national audit of cancer diagnosis. J Public Health. 2018 Jan 27; Available from: http://dx.doi.org/10.1093/pubmed/fdx188

25. Walter FM, Emery JD, Mendonca S, Hall N, Morris HC, Mills K, et al. Symptoms and patient factors associated with longer time to diagnosis for colorectal cancer: results from a prospective cohort study. Br J Cancer. 2016 Aug 4;115:533.

26. Walter FM, Rubin G, Bankhead C, Morris HC, Hall N, Mills K, et al. Symptoms and other factors associated with time to diagnosis and stage of lung cancer: a prospective cohort study. Br J Cancer. 2015 Mar 31;112(Suppl 1):S6–13.

For peer review only

27. Walter FM, Mills K, Mendonça SC, Abel GA, Basu B, Carroll N, et al. Symptoms and patient factors associated with diagnostic intervals for pancreatic cancer (SYMPTOM pancreatic study): a prospective cohort study. Lancet Gastroenterol Hepatol. 2016 Dec 1;1(4):298–306.

28. Din NU, Ukoumunne OC, Rubin G, Hamilton W, Carter B, Stapley S, et al. Age and gender variations in cancer diagnostic intervals in 15 cancers: analysis of data from the UK Clinical Practice Research Datalink. PLoS One. 2015;10(5):e0127717.

29. Redaniel MT, Martin RM, Ridd MJ, Wade J, Jeffreys M. Diagnostic intervals and its association with breast, prostate, lung and colorectal cancer survival in England: historical cohort study using the clinical practice research datalink. PloS One. 2015;10(5):e0126608.

30. Guldbrandt LM, Fenger-Grøn M, Rasmussen TR, Jensen H, Vedsted P. The role of general practice in routes to diagnosis of lung cancer in Denmark: a population-based study of general practice involvement, diagnostic activity and diagnostic intervals. BMC Health Serv Res. 2015;15(1):21.

31. Lyratzopoulos G, Saunders CL, Abel GA, McPhail S, Neal RD, Wardle J, et al. The relative length of the patient and the primary care interval in patients with 28 common and rarer cancers. Br J Cancer. 2015 Mar 3;112:S35.

32. Baughan P, O'Neill B, Fletcher E. Auditing the diagnosis of cancer in primary care: the experience in Scotland. Br J Cancer. 2009 Dec 3;101:S87.

33. Rubin G, Berendsen A, Crawford SM, Dommett R, Earle C, Emery J, et al. The expanding role of primary care in cancer control. Lancet Oncol. 2015;16(12):1231–72.

34. Green T, Atkin K, Macleod U. Cancer detection in primary care: insights from general practitioners. Br J Cancer. 2015;112(s1):S41.

Acknowledgements

The work was undertaken as part of the CRUK-PHE partnership and the ACE Programme, which is CRUK, Macmillan cancer support and NHS England supported. The authors would also like to thank Ruth Swann & Jess Fraser (CRUK-PHE partnership), Dave Chapman (ACE Programme, CRUK) and the National Cancer Diagnosis Audit.

Data for this study is based on patient-level information collected by the NHS, as part of the care and support of cancer patients. The data are collated, maintained and quality assured by the National Cancer Registration and Analysis Service, which is part of Public Health England

Funding statement

WH is co-PI and GR steering group chair of CanTest, a Cancer Research UK Catalyst Award [C8640/A23385].

Patient consent

Not required

Ethics

For peer review only

This study was exempt from gaining individual consent having obtained Section 251 approval from the UK Patient Advisory Group (PIAG) (now the Confidentiality Advisory Group, CAG), under Section 251 of the NHS Act 2006 (PIAG 03(a)/2001).

Conflicts of interests

The authors declare no conflicts of interests

Author statement

Conception and design of the work: CP, VP, KF, WH.

Analysis of the data: CP with input from VP

Contributions to the interpretation of the findings: CP, VP, KF, GR, WH.

All authors contributed to drafting the manuscript or revising it critically for important intellectual content and approved the final version submitted.

All authors have agreed to be accountable for all aspects of the work.

Data availability

The data for this study is collected and managed by Public Health England (PHE). The release of potentially identifiable data is managed through the Office for Data Release (ODR). The ODR provides a common governance framework for responding to requests to access PHE data, and is subject to strict confidentiality provisions in line with the requirements of the Common Law Duty of Confidentiality, the Data Protection Act 1998 (superseded by the General Data Protection Regulation (EU) 2016/679 which will take effect on 25 May 2018) and the 7 Caldicott principles. Applications to access this linked prescriptions data for patients with cancer should be directed through the ODR (odr@phe.gov.uk) and application forms are available on their website.

For peer review only

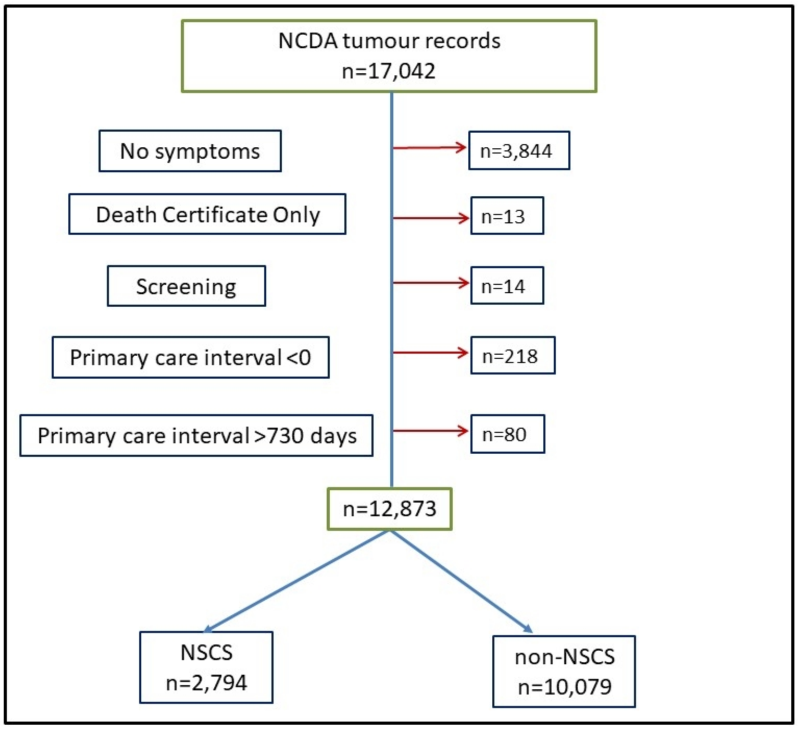

Figure 1: Data exclusions and allocation to analysis groups

81x60mm (300 x 300 DPI)

Cross-sectional study using primary care and cancer registration data to investigate cancer patients presenting with non-specific symptoms.

Supplementary Information

Table S1: Site groupings: ICD10 (International Classification of Disease version 10) codes

Site name	ICD10 codes (3 digit)
Brain & Central Nervous System (CNS)	C69-C72; C47
Breast	C50
Colorectal	C17-C21; C26
Gynaecology	C51-C58
Haematology	C81-C85; C88; C90-C93; C95-C96
Head & Neck	C00-C14; C30-C32; C73
Lung	C33-C34; C37-C38; C45
Sarcoma	C40-C41; C46; C48-C49
Skin	C43
Upper Gastrointestinal (GI)	C15-C16; C22-C25
Urology	C60-C68
Other	C74-C80

For peer review only

For peer review only - http://bmjopen.bmj.com/site/about/guidelines.xhtml

Table S2: Regression analysis results – associations (Odds Ratios – OR) with having a long primary care interval

	All		NSCS		Non-NSCS	
	Unadjusted OR (95%CI)	Adjusted OR (95%CI)	Unadjusted OR (95%CI)	Adjusted OR (95%CI)	Unadjusted OR (95%CI)	Adjusted OR (95%CI)
**NSCS**						
NSCS	**1.59 (1.46-1.73)**	**1.24 (1.12-1.36)**				
Non-NSCS	Reference	Reference				
**Age group**						
0-24	1.34 (0.97-1.86)	0.95 (0.67-1.34)	1.91 (0.91-4.00)	1.56 (0.71-3.44)	1.23 (0.86-1.78)	0.79 (0.53-1.17)
25-44	**0.78 (0.67-0.92)**	1.08 (0.90-1.29)	1.22 (0.81-1.85)	1.37 (0.88-2.12)	**0.76 (0.64-0.91)**	1.02 (0.84-1.25)
45-59	**0.88 (0.78-0.98)**	1.08 (0.95-1.21)	1.06 (0.83-1.37)	1.15 (0.89-1.50)	**0.85 (0.75-0.97)**	1.05 (0.92-1.21)
60-69	Reference	Reference	Reference	Reference	Reference	Reference
70-79	1.04 (0.94-1.14)	0.98 (0.88-1.09)	0.96 (0.78-1.18)	0.94 (0.76-1.16)	1.05 (0.94-1.17)	0.98 (0.87-1.11)
80+	1.10 (0.99-1.21)	0.97 (0.87-1.08)	0.93 (0.76-1.15)	0.86 (0.69-1.07)	1.12 (0.99-1.26)	1.01 (0.88-1.15)
**Sex**						
Male	Reference	Reference	Reference	Reference	Reference	Reference
Female	**0.79 (0.74-0.85)**	1.09 (1.00-1.19)	1.05 (0.90-1.22)	1.12 (0.95-1.32)	**0.72 (0.67-0.78)**	1.08 (0.98-1.20)
**Deprivation**						
1 - least deprived	Reference	Reference	Reference	Reference	Reference	Reference
2	**1.12 (1.01-1.25)**	1.12 (1.00-1.25)	1.04 (0.82-1.31)	1.04 (0.82-1.32)	**1.14 (1.01-1.28)**	1.14 (1.00-1.30)
3	1.09 (0.98-1.21)	1.05 (0.94-1.17)	1.07 (0.85-1.35)	1.05 (0.82-1.32)	1.08 (0.96-1.22)	1.04 (0.92-1.18)
4	**1.18 (1.06-1.32)**	1.10 (0.98-1.23)	1.18 (0.93-1.49)	1.15 (0.90-1.46)	**1.16 (1.02-1.31)**	1.08 (0.94-1.23)
5 - most deprived	**1.19 (1.06-1.34)**	1.08 (0.96-1.23)	1.18 (0.92-1.51)	1.13 (0.88-1.45)	**1.17 (1.03-1.34)**	1.06 (0.92-1.22)
**Comorbidities**						
0	Reference	Reference	Reference	Reference	Reference	Reference
1	**1.20 (1.10-1.32)**	**1.13 (1.02-1.25)**	**1.27 (1.03-1.55)**	**1.38 (1.11-1.72)**	**1.17 (1.05-1.30)**	1.06 (0.94-1.19)
2	**1.21 (1.09-1.33)**	1.11 (0.99-1.25)	1.18 (0.95-1.46)	**1.30 (1.03-1.65)**	**1.19 (1.06-1.33)**	1.06 (0.93-1.21)
3+	**1.46 (1.31-1.61)**	**1.26 (1.11-1.42)**	**1.29 (1.04-1.61)**	**1.44 (1.12-1.84)**	**1.48 (1.32-1.66)**	**1.20 (1.04-1.38)**
**Route to diagnosis**						
Emergency presentation	**3.06 (2.78-3.37)**	**2.08 (1.87-2.32)**	**1.76 (1.46-2.13)**	**1.66 (1.36-2.03)**	**3.40 (3.03-3.82)**	**2.23 (1.95-2.54)**
GP referral	**1.78 (1.63-1.95)**	**1.44 (1.31-1.58)**	**1.32 (1.07-1.62)**	**1.28 (1.04-1.58)**	**1.84 (1.66-2.04)**	**1.48 (1.33-1.64)**

For peer review only - http://bmjopen.bmj.com/site/about/guidelines.xhtml

Inpatient	**2.65 (2.03-3.45)**	**2.04 (1.55-2.67)**	1.26 (0.74-2.13)	1.18 (0.69-2.03)	**3.15 (2.31-4.28)**	**2.40 (1.75-3.30)**
Outpatient	**3.40 (2.95-3.91)**	**2.47 (2.13-2.86)**	**1.88 (1.40-2.52)**	**1.71 (1.26-2.31)**	**3.82 (3.25-4.48)**	**2.73 (2.31-3.23)**
Two Week Wait	Reference	Reference	Reference	Reference	Reference	Reference
Unknown	**2.90 (2.32-3.62)**	**2.69 (2.13-3.39)**	**1.99 (1.20-3.31)**	**1.85 (1.10-3.12)**	**3.08 (2.41-3.95)**	**2.91 (2.24-3.76)**
**Stage**						
1	Reference	Reference	Reference	Reference	Reference	Reference
2	**0.88 (0.78-0.99)**	0.93 (0.81-1.05)	0.89 (0.65-1.22)	0.94 (0.68-1.30)	**0.83 (0.73-0.95)**	0.93 (0.81-1.07)
3	**1.20 (1.07-1.36)**	0.96 (0.85-1.09)	0.97 (0.72-1.30)	1.04 (0.76-1.41)	**1.17 (1.03-1.34)**	0.94 (0.81-1.09)
4	**1.76 (1.58-1.95)**	1.06 (0.94-1.20)	1.21 (0.93-1.58)	1.14 (0.86-1.51)	**1.76 (1.56-1.98)**	1.03 (0.90-1.18)
Unknown/other	**1.81 (1.63-2.02)**	1.08 (0.95-1.23)	1.31 (1.00-1.72)	1.22 (0.90-1.64)	**1.79 (1.59-2.01)**	1.04 (0.90-1.20)
**Site**						
Brain & CNS$	**2.86 (2.12-3.84)**	**2.27 (1.65-3.12)**	**17.42 (2.29-132.50)**	**12.57 (1.61-97.95)**	**3.11 (2.26-4.27)**	**2.19 (1.55-3.08)**
Breast	**0.33 (0.28-0.39)**	**0.47 (0.39-0.56)**	1.66 (0.63-4.40)	1.58 (0.58-4.31)	**0.38 (0.32-0.46)**	**0.48 (0.39-0.60)**
Colorectal	Reference	Reference	Reference	Reference	Reference	Reference
Gynaecology	**0.75 (0.63-0.89)**	**0.81 (0.67-0.98)**	0.88 (0.63-1.22)	0.84 (0.59-1.20)	0.81 (0.65-1.01)	0.82 (0.65-1.04)
Haematology	**1.46 (1.25-1.70)**	**1.38 (1.17-1.62)**	**1.39 (1.08-1.79)**	1.24 (0.94-1.63)	**1.64 (1.35-2.01)**	**1.49 (1.20-1.83)**
Head & neck	0.95 (0.78-1.16)	1.10 (0.89-1.35)	1.63 (0.51-5.17)	1.38 (0.43-4.44)	1.12 (0.89-1.40)	1.15 (0.91-1.46)
Lung	**1.89 (1.65-2.16)**	**1.82 (1.58-2.11)**	**1.66 (1.31-2.11)**	**1.55 (1.21-1.99)**	**2.22 (1.86-2.65)**	**1.98 (1.64-2.38)**
Other	**1.59 (1.26-2.00)**	1.26 (0.98-1.62)	1.24 (0.87-1.77)	0.97 (0.65-1.44)	**1.99 (1.46-2.71)**	**1.57 (1.13-2.20)**
Sarcoma	**1.52 (1.08-2.12)**	**1.61 (1.13-2.28)**	1.16 (0.55-2.47)	1.08 (0.50-2.33)	**1.85 (1.26-2.71)**	**1.85 (1.24-2.76)**
Skin	**0.42 (0.35-0.52)**	**0.57 (0.46-0.71)**	1.16 (0.07-18.63)	1.33 (0.08-21.86)	**0.50 (0.40-0.63)**	**0.60 (0.47-0.75)**
Upper GI@	1.13 (0.98-1.31)	1.05 (0.90-1.23)	1.15 (0.92-1.43)	1.04 (0.83-1.31)	1.17 (0.96-1.43)	1.08 (0.87-1.33)
Urology	1.02 (0.90-1.16)	**1.26 (1.10-1.45)**	1.26 (0.96-1.65)	**1.39 (1.04-1.85)**	1.16 (0.98-1.37)	**1.29 (1.08-1.53)**

* Adjusted model – adjusted for NSCS (in overall cohort only), age, sex, deprivation, comorbidities, route, stage, site

$ - Central Nervous System

@ - Upper Gastrointestinal

For peer review only - http://bmjopen.bmj.com/site/about/guidelines.xhtml

STROBE 2007 (v4) Statement—Checklist of items that should be included in reports of *cross-sectional studies*

Section/Topic	Item #	Recommendation	Reported on page #
**Title and abstract**	1	(*a*) Indicate the study's design with a commonly used term in the title or the abstract	1
		(*b*) Provide in the abstract an informative and balanced summary of what was done and what was found	1
**Introduction**			
Background/rationale	2	Explain the scientific background and rationale for the investigation being reported	3
Objectives	3	State specific objectives, including any prespecified hypotheses	3
**Methods**			
Study design	4	Present key elements of study design early in the paper	3
Setting	5	Describe the setting, locations, and relevant dates, including periods of recruitment, exposure, follow-up, and data collection	N/A
Participants	6	(*a*) Give the eligibility criteria, and the sources and methods of selection of participants	N/A
Variables	7	Clearly define all outcomes, exposures, predictors, potential confounders, and effect modifiers. Give diagnostic criteria, if applicable	4&5
Data sources/ measurement	8*	For each variable of interest, give sources of data and details of methods of assessment (measurement). Describe comparability of assessment methods if there is more than one group	3
Bias	9	Describe any efforts to address potential sources of bias	N/A in methods
Study size	10	Explain how the study size was arrived at	4
Quantitative variables	11	Explain how quantitative variables were handled in the analyses. If applicable, describe which groupings were chosen and why	5
Statistical methods	12	(*a*) Describe all statistical methods, including those used to control for confounding	5
		(*b*) Describe any methods used to examine subgroups and interactions	5
		(*c*) Explain how missing data were addressed	4
		(*d*) If applicable, describe analytical methods taking account of sampling strategy	N/A
		(*e*) Describe any sensitivity analyses	5
**Results**			

For peer review only

Participants	13*	(a) Report numbers of individuals at each stage of study—eg numbers potentially eligible, examined for eligibility, confirmed eligible, included in the study, completing follow-up, and analysed	5
		(b) Give reasons for non-participation at each stage	5
		(c) Consider use of a flow diagram	5
Descriptive data	14*	(a) Give characteristics of study participants (eg demographic, clinical, social) and information on exposures and potential confounders	7
		(b) Indicate number of participants with missing data for each variable of interest	
Outcome data	15*	Report numbers of outcome events or summary measures	7&8
Main results	16	(*a*) Give unadjusted estimates and, if applicable, confounder-adjusted estimates and their precision (eg, 95% confidence interval). Make clear which confounders were adjusted for and why they were included	10&11
		(*b*) Report category boundaries when continuous variables were categorized	7&8, 10&11
		(*c*) If relevant, consider translating estimates of relative risk into absolute risk for a meaningful time period	N/A
Other analyses	17	Report other analyses done—eg analyses of subgroups and interactions, and sensitivity analyses	9
**Discussion**			
Key results	18	Summarise key results with reference to study objectives	12
Limitations	19	Discuss limitations of the study, taking into account sources of potential bias or imprecision. Discuss both direction and magnitude of any potential bias	12
Interpretation	20	Give a cautious overall interpretation of results considering objectives, limitations, multiplicity of analyses, results from similar studies, and other relevant evidence	12&13
Generalisability	21	Discuss the generalisability (external validity) of the study results	13
**Other information**			
Funding	22	Give the source of funding and the role of the funders for the present study and, if applicable, for the original study on which the present article is based	16

*Give information separately for cases and controls in case-control studies and, if applicable, for exposed and unexposed groups in cohort and cross-sectional studies.

Note: An Explanation and Elaboration article discusses each checklist item and gives methodological background and published examples of transparent reporting. The STROBE checklist is best used in conjunction with this article (freely available on the Web sites of PLoS Medicine at http://www.plosmedicine.org/, Annals of Internal Medicine at http://www.annals.org/, and Epidemiology at http://www.epidem.com/). Information on the STROBE Initiative is available at www.strobe-statement.org.

For peer review only
